# CIB2 interacts with TMC1 and TMC2 and is essential for mechanotransduction in auditory hair cells

**DOI:** 10.1038/s41467-017-00061-1

**Published:** 2017-06-29

**Authors:** Arnaud P. J. Giese, Yi-Quan Tang, Ghanshyam P. Sinha, Michael R. Bowl, Adam C. Goldring, Andrew Parker, Mary J. Freeman, Steve D. M. Brown, Saima Riazuddin, Robert Fettiplace, William R. Schafer, Gregory I. Frolenkov, Zubair M. Ahmed

**Affiliations:** 1 Department of Otorhinolaryngology Head & Neck Surgery, School of Medicine, University of Maryland, Baltimore, MD 21201 USA; 2 Division of Neurobiology, MRC Laboratory of Molecular Biology, Cambridge Biomedical Campus, Cambridge, CB2 0QH UK; 3 Department of Physiology, College of Medicine, University of Kentucky, Lexington, KY 40536-0298 USA; 4 MRC Harwell Institute, Mammalian Genetics Unit, Harwell Campus, Oxfordshire, OX11 0RD UK; 5 Department of Neuroscience, School of Medicine, University of Wisconsin, Madison, WI 53706 USA

## Abstract

Inner ear hair cells detect sound through deflection of stereocilia, the microvilli-like projections that are arranged in rows of graded heights. Calcium and integrin-binding protein 2 is essential for hearing and localizes to stereocilia, but its exact function is unknown. Here, we have characterized two mutant mouse lines, one lacking calcium and integrin-binding protein 2 and one carrying a human deafness-related *Cib2* mutation, and show that both are deaf and exhibit no mechanotransduction in auditory hair cells, despite the presence of tip links that gate the mechanotransducer channels. In addition, mechanotransducing shorter row stereocilia overgrow in hair cell bundles of both *Cib2* mutants. Furthermore, we report that calcium and integrin-binding protein 2 binds to the components of the hair cell mechanotransduction complex, TMC1 and TMC2, and these interactions are disrupted by deafness-causing *Cib2* mutations. We conclude that calcium and integrin-binding protein 2 is required for normal operation of the mechanotransducer channels and is involved in limiting the growth of transducing stereocilia.

## Abstract (web summary)

Inner ear hair cells detect sound through deflection of stereocilia that harbor mechanically-gated channels. Here the authors show that protein responsible for Usher syndrome, CIB2, interacts with these channels and is essential for their function and hearing in mice.

## Introduction

Mammalian hearing relies on stereocilia, the actin-filled mechanosensitive projections at the apical surface of sensory hair cells in the organ of Corti. In each hair cell, stereocilia are organized in a hair bundle with rows of precisely determined increasing heights. This “staircase” architecture of the bundle is conserved across all vertebrate hair cells and is essential for normal hearing^1^. It allows effective pulling of the tip links between stereocilia of neighboring rows^2^ and mechanical gating of the transducer channels that are located at the lower ends of the tip links, i.e., at the tips of shorter but not tallest rows of stereocilia^3^. Mature tip links are formed by two cadherin molecules, protocadherin 15 and cadherin 23^4, 5^. At the lower end of the tip link, protocadherin 15 may interact with TMC1 and TMC2^6^, the proposed core components of the mechano-electrical transduction (MET) complex^7^. It is still a subject of debate whether TMC1 and TMC2 could form an ion channel and represent the pore-forming subunits of the MET channel^8–10^. However, these transmembrane proteins have been demonstrated to be essential for the MET complex, in addition to the other presumably auxiliary subunits LHFPL5 and TMIE^7,11–13^. Some of these or additional components of the MET machinery should have Ca^2+^-binding element(s) to account for the multiple Ca^2+^ effects on the MET current^14–18^. However, the molecular identity of Ca^2+^-sensitive component(s) of the MET machinery is still unknown.

We have previously identified calcium and integrin-binding protein 2 (CIB2) as a novel protein associated with nonsyndromic deafness (at *DFNB48* locus) and Usher syndrome type I in humans^19^. CIB2 belongs to a family of four known proteins, CIB1 through CIB4, that contain four helix-loop-helix domains, also called EF hand domains (EF1-EF4)^20^. The first EF hand domain of CIB1 has been shown to be inactive and does not bind Ca^2+^, while the remaining three EF hand domains do and are thought to mediate intracellular Ca^2+^ signaling^20^. Most of the work has been done on the functional characterization of the CIB1 protein. CIB1 is implicated in many functions, such as thrombosis, spermatogenesis, cell proliferation, apoptosis, cytoskeleton organization, angiogenesis, tumor growth, and pathological cardiac hypertrophy^21–26^. CIB2 contains only three EF hand domains and is able to bind calcium through the second and third domains^27^. Fluorescence resonance energy transfer (FRET) measurements confirmed the changes in CIB2 conformation upon Ca^2+^ binding^27^. We previously showed that CIB2 is localized to the stereocilia of rodent hair cells^19^. Here, we show that CIB2 interacts with the MET channel components TMC1 and TMC2, is essential for MET function and regulating the length of transducing shorter row stereocilia in mammalian auditory hair cells.

## Results

### Expression and generation of *Cib2*-mutant mouse strains

**Fig. 1:**
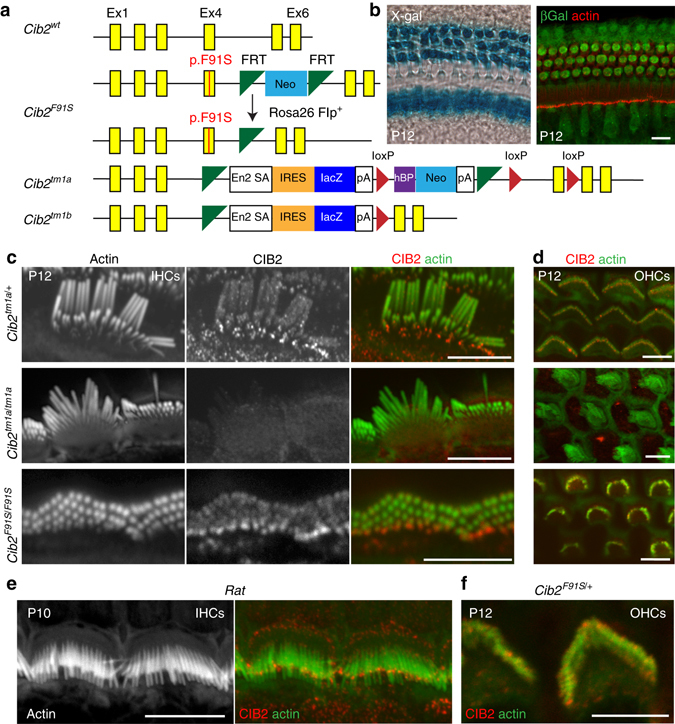
CIB2 is localized in the auditory hair cell stereocilia. **a** Structure of the wild-type, the *Cib2* ^*F91S*^ and the *Cib2* ^*tm1a(EUCOMM)Wtsi*^ (*Cib2* ^*tm1a*^, EUCOMM) alleles. **b** X-gal staining to document the *Cib2* locus activity in the organ of Corti of P12 *Cib2* ^*tm1a/+*^ mice (*left*). Immunolocalization of β-galactosidase (*green*) in *Cib* ^*2tm1a/+*^ mouse cochlea at P12 (*right*). The cochlear actin is labeled with rhodamine–phalloidin (*red*). **c**, **d** Confocal images of IHC (**c**) and OHC (**d**) stereocilia bundles immunostained with CIB2 antibody (*middle panels* in **c** and *red* in the *merge panels*), and actin was labeled with phalloidin (*left panels* in **c** and *green* in the *merge panels*) in the control *Cib2* ^*tm1a/+*^, *Cib2* ^*tm1a/tm1a*^, and *Cib2* ^*F91S/F91S*^ mice at P12. Note that *Cib2* truncation causes disappearance of CIB2 protein from stereocilia, while p.F91S mutation does not. **e** CIB2 localization in the rat IHC stereocilia at P10. **f** CIB2 localization at P12 in *Cib2* ^*F91S/+*^ OHCs. Scale bars: **b** 10 μm, **c**–**f** 5 μm

To determine the role of CIB2 in the inner ear, we generated a p.F91S missense mutation knockin mouse (*Cib2* ^*F91S*^) (Fig. 1a), which corresponds to the most prevalent *CIB2* allele found in human families with nonsyndromic deafness^19^. We also used *Cib2* ^*tm1a*^ mutant mice. These mice carry a gene trap cassette with a lacZ reporter between exons 3 and 4 (Fig. 1a). The gene trap leads to the translation of a truncated protein consisting of only the first 66 amino-acids of CIB2. Homozygous *Cib2* ^*tm1a/tm1a*^ and *Cib2* ^*F91S/F91S*^ mutant mice are fertile and appear healthy. *Cib2* ^*tm1a*^ mice were crossed with ubiquitous Cre expressers (C57BL/6NTac-Tg(ACTB-cre)^3Mrt^/H) to delete the neomycin cassette and exon 4 of *Cib2* (*Cib2* ^*tm1b*^; Fig. 1a). *Cib2* is ubiquitously expressed (Fig. 1b, Supplementary Fig. 1a)^19^. Phenotyping of multiple organs from *Cib2* ^*tm1b*^ mice, missing the neo cassette (Fig. 1a), revealed abnormal voluntary movements, circulating high-density lipoprotein-cholesterol level, and heart left ventricle morphology, in addition to the loss of the startle response and elevated auditory thresholds (Supplementary Table 1). X-gal staining and β-gal immunostaining revealed that *Cib2* is highly expressed in sensory hair cells of both the organ of Corti and the vestibular system. We did not observe any changes of *Cib2* expression during the first postnatal week or differences in *Cib2* expression along the length of the cochlea (Fig. 1b, Supplementary Fig. 1b).

Using an antibody directed against the N-terminus, wild-type CIB2 was observed in the stereocilia and at the tips of surrounding microvilli of the auditory hair cells of control heterozygous mice (Fig. 1c, *top*). We detected CIB2 in the stereocilia of cochlear hair cells at P12 and as early as P5 (Fig. 1c, Supplementary Fig. 2a, b). Similar labeling was observed in rat auditory hair cells (Fig. 1e). We were not able to detect CIB2 immunolabeling in the stereocilia at earlier ages, probably due to relatively low affinity of our antibodies. According to the SHIELD^28^ database, *Cib2* is expressed in the organ of Corti hair cells as early as embryonic day 16. We also detected prominent lacZ reporter expression driven by *Cib2* promoter at postnatal day 0. Furthermore, we demonstrated that exogenous CIB2 is properly targeted to the auditory hair cell stereocilia as early as P2-P3^19^. In the inner hair cells (IHCs) of control heterozygous animals, CIB2 was distributed along the length of stereocilia and accumulated at the tips of the shortest (but still mechanotransducing^3^) stereocilia (Fig. 1c, *top*). In the outer hair cells (OHCs), we observed punctate labeling along the length of stereocilia, including labeling at the very tips of OHC stereocilia (Fig. 1d, *top* and Fig. 1f). CIB2 immuno-labeling was specific, because it completely disappeared from stereocilia bundles of *Cib2* ^*tm1a/tm1a*^ mice (Fig. 1c, d, *middle*). Therefore, our new immunolabeling data are consistent with the previously hypothesized role of CIB2 in mechanotransduction^19^, although its additional role outside the MET complex cannot be excluded.

Since our antibodies were raised against N-terminus (amino acid 1–99), they may recognize not only wild-type CIB2 but also truncated CIB2 in *Cib2* ^*tm1a/tm1a*^ mice and mutated CIB2 in *Cib2* ^*F91S/F91S*^ mice. In contrast to the absence of truncated CIB2 in *Cib2* ^*tm1a/tm1a*^ stereocilia (Fig. 1c, d, *middle*), the p.F91S point mutation does not affect CIB2 labeling in the stereocilia of *Cib2* ^*F91S/F91S*^ mice (Fig. 1c, d, *bottom*). This is consistent with our previous observation that exogenous CIB2 protein with p.F91S variant is properly targeted to the stereocilia in the cultured vestibular sensory epithelia^29^. Thus, we concluded that p.F91S mutation does not disrupt localization of CIB2 in hair cell stereocilia.

### *Cib2* mutant mice are deaf

**Fig. 2:**
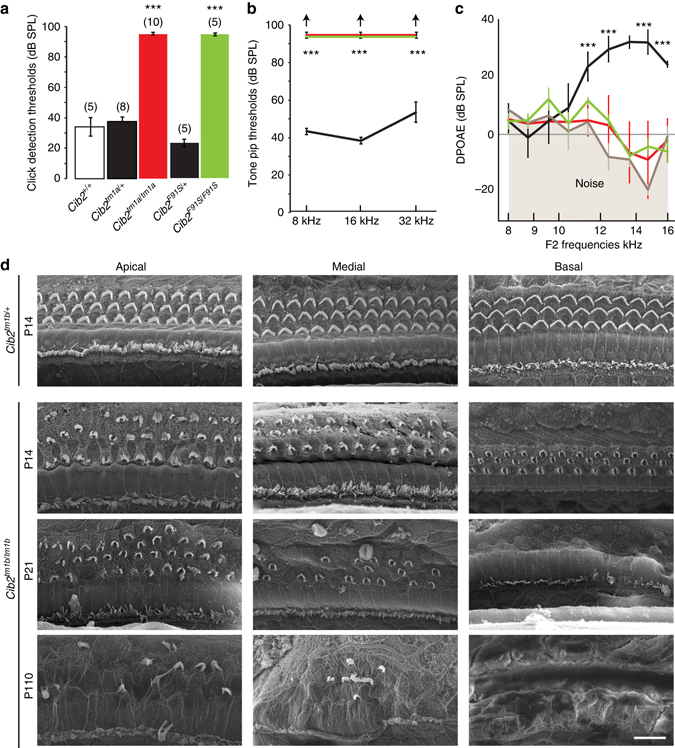
CIB2 deficiency leads to profound sensorineural hearing loss and degeneration of the organ of Corti. **a**, **b** ABR thresholds to broadband clicks (**a**) and tone-pips with frequencies of 8 kHz, 16 kHz, and 32 kHz (**b**) in wild-type (*open bar*), *Cib2* ^*tm1a/+*^, *Cib2* ^*F91S/+*^ (*black*), *Cib2* ^*tm1a/tm1a*^ (*red*), and *Cib2* ^*F91S/F91S*^ (*green*) mice at P16. Number of animals in each group is shown in parentheses. The same animals were tested with clicks and tone pips. **c** DPOAEs of control (*black*; *n* = 13), *Cib2* ^*tm1a/tm1a*^ (*red*; *n* = 10), and *Cib2* ^*F91S/F91S*^ (*green*; *n* = 5) mice at P16-P18. Noise floor is shown in *grey*. All data in **a**–**c** are shown as Mean ± SEM (***p < 0.001). **d** SEM images of the organ of Corti at the apical, medial and basal turns of the cochlea in the control, and *Cib2* ^*tm1b/tm1b*^ mice at P14, P21 and P110. Scale bar: 10 μm

To characterize hearing function, we measured auditory-evoked brainstem responses (ABR) in *Cib2* mutant and control littermate mice at P16. Normal ABR waveforms and thresholds were observed in wild-type and heterozygous mice. However, both *Cib2* ^*tm1a/tm1a*^ and *Cib2* ^*F91S/F91S*^ homozygous mice did not respond to click or tone burst stimuli of 100 dB sound pressure level (SPL), indicating that they are profoundly deaf (Fig. 2a, b), recapitulating human DFNB48/USH1J deafness. We also recorded distortion product otoacoustic emission (DPOAE), a by-product of cochlear amplification that depends on the integrity of OHCs. At P16-P18, wild-type and *Cib2* ^*tm1a/+*^ and *Cib2* ^*F91S/+*^ heterozygous mice generated apparently normal and similar DPOAEs. However, in both *Cib2* mutant mice, DPOAEs were indiscernible from the noise floor (Fig. 2c). Taken together with ABRs, these results suggest that hearing loss in CIB2 mutant mice is caused by peripheral (cochlear) deficiencies.

Despite prominent hearing loss, both *Cib2* ^*tm1a/tm1a*^ and *Cib2* ^*F91S/F91S*^ mice display no obvious indications of vestibular dysfunction, such as circling, hyperactivity, or head bobbing. The absence of an overt vestibular phenotype in both *Cib2* mutants indicates that in contrast to humans, CIB2 function is likely to be redundant in mouse vestibular hair cells. To test this possibility, we used quantitative reverse transcription polymerase chain reaction to investigate the expression of the *Cib* gene family (*Cib1-4*) in cochlear and vestibular sensory epithelia at P12. In the control mice, the most dramatic difference between vestibular and cochlear tissues was observed for *Cib3*, which showed an almost 8-fold higher expression in the vestibular samples (Supplementary Fig. 3a). In *Cib2* ^*tm1a/tm1a*^ mice, CIB2 deficiency resulted in significant upregulation of *Cib1* in the cochlea (Supplementary Fig. 3b) and slight, not statistically significant, upregulation of *Cib3* in the vestibular periphery (Supplementary Fig. 3b). Mouse CIB2 is 61% identical and 78% similar to CIB3 and both proteins share identical domain structure with three EF hand domains. Therefore, functional redundancy between these proteins is plausible. Substantially larger expression of *Cib3* in the vestibular periphery (Supplementary Fig. 3b) may account for the relative insensitivity of the vestibular end organs to the loss of *Cib2* compared to the cochlea.

Surprisingly, we did not observe any differences in ABR or DPOAE between *Cib2* ^*tm1a/tm1a*^ mice that have no CIB2 in the stereocilia and *Cib2* ^*F91S/F91S*^ mice that still have mutant CIB2 at the stereocilia tips. We conclude that, while the F91S mutation does not affect protein localization, it does cause loss of CIB2 function. As such, the *Cib2* ^*F91S/F91S*^ mice represent a good experimental model to study functional effects of CIB2 interactions with other stereocilia proteins.

### Morphological changes in the organ of Corti of *cib2* mutants

Gross morphology and cytoarchitecture of the organs of Corti in both *Cib2* ^*tm1a/tm1a*^ and *Cib2* ^*F91S/F91S*^ mice appear normal at postnatal day 0 (Fig. 2d, Supplementary Fig. 4a–c). However, by P16-18, OHCs of both *Cib2* mutant mice started to degenerate in the mid-basal part of the cochlea, which was exacerbated by P27 (Fig. 2d, Supplementary Fig. 4b, c). IHCs remain largely intact, though some of them were missing around P27 (Fig. 2d, Supplementary Fig. 4b, c). In P110 mice, OHCs completely degenerated and only few IHCs survived (Fig. 2d). The loss of sensory hair cells was followed by the progressive degeneration of spiral ganglion neurons (Supplementary Fig. 5).

### Stereocilia bundle morphology in the auditory hair cells of *Cib2* mutants

**Fig. 3:**
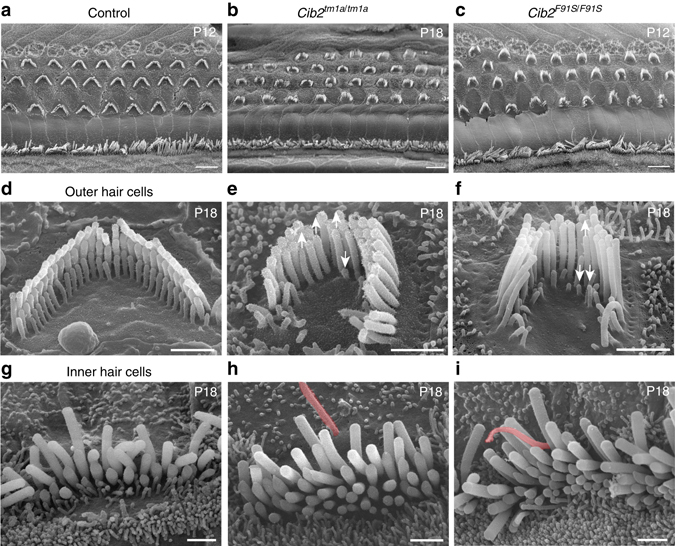
CIB2 deficiency disrupts stereocilia bundle morphology of the auditory hair cells. SEM images of the organs of Corti (**a**–**c**), as well as OHC (**d**–**f**) and IHC (**g**–**i**) stereocilia bundles from control heterozygous (**a**, **d**, **g**), *Cib2* ^*tm1a/tm1a*^ (**b**, **e**, **h**), and *Cib2* ^*F91S/F91S*^ (**c**, **f**, **i**) mice at the mid-cochlear location. Over-grown and retracted stereocilia are shown by *arrows up* and *down*, respectively. The IHC kinocilium does not regress properly in *Cib2* mutant mice (*pink*). Scale bars: **a**–**c** 5 µm, (**d**–**i**) 1 µm

Next, we evaluated the morphology of the stereocilia bundles by scanning electron microscopy (SEM). Although there was no loss of either OHCs or IHCs at P12-18 in the middle of the cochlea (Fig. 3a–c), we observed identical abnormalities in the stereocilia bundles of both *Cib2* ^*tm1a/tm1a*^ and *Cib2* ^*F91S/F91S*^ mice. The mutant OHC bundles had a horseshoe shape and, most importantly, a disrupted staircase architecture. Instead of building precise 2nd and 3rd rows of stereocilia in the bundle (Fig. 3d), these cells often over-grew the second row of stereocilia, while the third rows were either over-grown (Fig. 3e, f, *arrows up*) or retracted (Fig. 3e, f, *arrows down*). In contrast to OHCs, the staircase arrangement of IHC stereocilia was still present in both *Cib2* ^*tm1a/tm1a*^ and *Cib2* ^*F91S/F91S*^ mice (Fig. 3g–i). However, in mutant IHCs, the 3rd and 4th row stereocilia were abnormally thick and the kinocilium failed to regress properly (Fig. 3h, i), which was confirmed with tubulin labeling (Supplementary Fig. 6a).

**Fig. 4:**
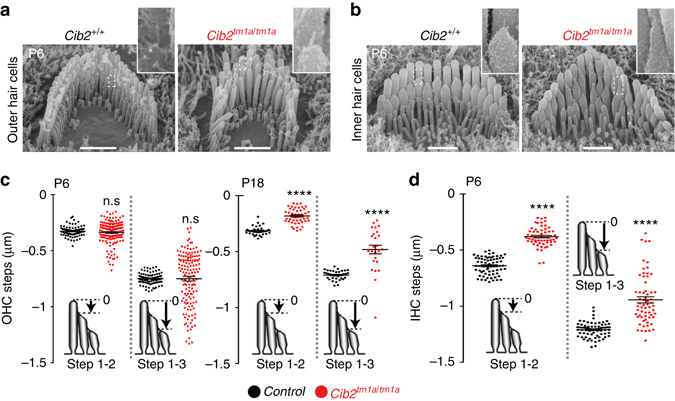
CIB2 deficiency leads to over-elongation of the transducing shorter row stereocilia in the auditory hair cells. **a**, **b** SEM images of OHC (**a**) and IHC (**b**) bundles from wild-type (*left*) and *Cib2* ^*tm1a/tm1a*^ (*right*) littermate mice at P6 at the same cochlear location in the middle of the apical turn. *Insets* show magnified images of stereocilia tips and stereocilia links in the areas indicated by *dashed boxes*. **c**, **d** Quantification of the step height differences between the 1st (longest) and 2nd and between the 1st and 3rd rows of stereocilia (illustrated on the *inset cartoons*) in the OHC (**c**) and IHC (**d**) bundles at P6 (**c**, *left* and **d**) and P18 (**c**, *right*). Each *dot* represents one pair of stereocilia. Mean ± SEM values are also shown. We were not able to quantify the staircase morphology in the IHCs at P18 because mouse IHCs are very fragile at this age and their bundles lose cohesiveness after dissection. Number of cells: IHCs, wild-type, *n* = 6; *Cib2* ^*tm1a/tm1a*^, *n* = 6; OHCs at P6, wild-type, *n* = 9; *Cib2* ^*tm1a/tm1a*^, *n* = 18; OHCs at P18, wild-type, *n* = 10; *Cib2* ^*tm1a/tm1a*^, *n* = 10. In all panels, asterisks indicate statistical significance of the difference between the data from mutant and wild-type cells. NS, not significant; ****p < 0.0001 (ANOVA). Scale bars: 1 μm

**Fig. 5:**
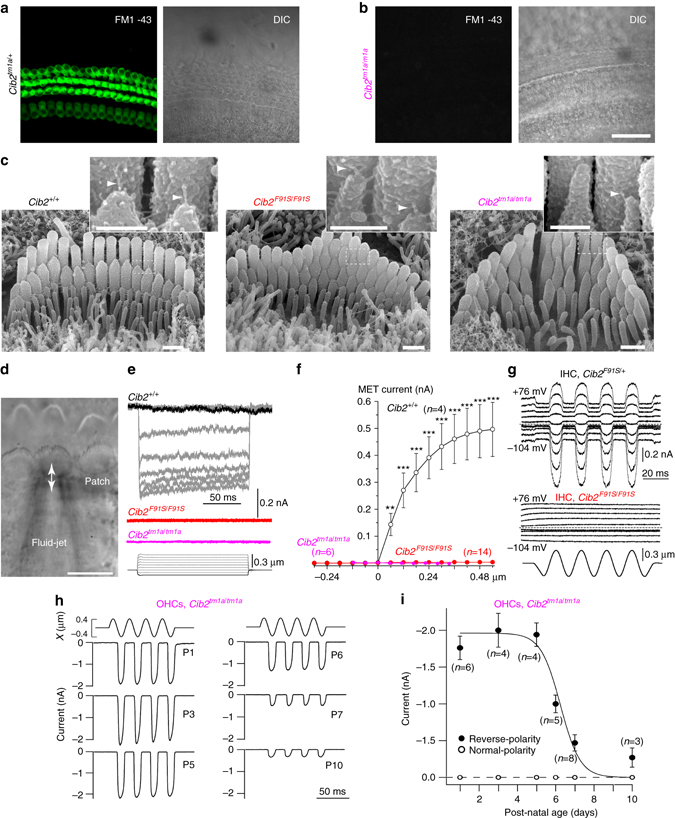
CIB2 is essential for mechanotransduction in the auditory hair cells. **a**, **b** Maximum intensity projections of Z-stacks of confocal fluorescent images (*left*) and corresponding DIC images (*right*) of control (**a**) and *Cib2* ^*tm1a/tm1a*^ (**b**) cultured organ of Corti explants imaged after exposure to 3 μM of FM1-43 for 10 s. The samples were dissected at P5 and kept 2 days in vitro (P5 + 2*div*). Scale bar: 20 µm. **c** SEM images of IHCs in acutely isolated organ of Corti explants from wild-type (*left*), *Cib2* ^*F91S/F91S*^ (*middle*), and *Cib2* ^*tm1a/tm1a*^ (*right*) mice. *Insets* show the tip links at high magnification in the areas indicated by *dashed boxes*. Scale bars are 0.5 µm and 200 nm in the *insets* (**d**). Experimental setup for MET current recordings. Positive pressure in fluid-jet deflects hair bundle toward kinocilium and activates “conventional” MET channels gated by tip link tension, while negative pressure closes these channels but may activate “reverse-polarity” currents at certain conditions^51, 52^. Scale bar is 10 µm (**e**, **f**) MET current traces (**e**) and average MET current (Mean ± SEM) (**f**) to the graded deflections of the hair bundles with fluid-jet (*bottom traces* in **e**) in wild-type (*black* and *grey*), *Cib2* ^*F91S/F91S*^ (red), and *Cib2* ^*tm1a/tm1a*^ (*magenta*) IHCs. Statistical significance is indicated with *asterisks*: **p < 0.01, ***p < 0.001 (Student’s *t*-test). **g** MET responses produced by a sinusoidal fluid-jet stimulus (*bottom*) in control *Cib2* ^*F91S/+*^ (*top traces*) and *Cib2* ^*F91S/F91S*^ (*middle traces*) IHCs at different holding potential ranging from −104 to +76 mV (indicated by the *traces*). Age of the IHCs in **e**–**g**: P4–P7. **h** Reverse-polarity current in the OHCs of *Cib2* ^*tm1a/tm1a*^ mice at different developmental ages indicated by the traces. Note that the current is activated by negative but not positive bundle deflections. **i** Average values (Mean ± SEM) of normal and reverse-polarity currents in *Cib2* ^*tm1a/tm1a*^ OHCs at different ages. Number of cells is indicated by each data point.

To explore the mechanisms of stereocilia growth dysregulation, we quantified the morphology of the OHC and IHC bundles in wild-type and *Cib2* ^*tm1a/tm1a*^ littermates at the same mid-apical location. The staircase morphology of the *Cib2* ^*tm1a/tm1a*^ OHC bundles showed severe disruption. At P6, this disruption was evident as an increased variability in heights of the shorter row stereocilia in *Cib2* ^*tm1a/tm1a*^ OHCs compared to wild-type OHCs, especially in the 3rd row, where individual stereocilium either over-grew or disassembled (Fig. 4a). At P6, we observed almost exclusively an overgrowth of the remaining shorter row stereocilia in the *Cib2* ^*tm1a/tm1a*^ OHCs (Fig. 4a). Similarly, as early as P6, *Cib2* ^*tm1a/tm1a*^ IHCs exhibited abnormally elongated tips of the 2nd and 3rd row stereocilia (Fig. 4b). A similar stereocilia phenotype with elongated tips was also observed in *Cib2* ^*F91S/F91S*^ IHCs (Fig. 5c). Using SEM images from the “back” side of the bundle, we measured the total height of individual IHC stereocilia in the tallest (1st) row of the bundle and found no differences between wild-type and *Cib2* ^*tm1a/tm1a*^ mice (2.08 ± 0.04 μm, number of analyzed stereocilia/cells *n* = 51/6; and 2.16 ± 0.14 μm, *n* = 41/6, correspondingly). However, measurements of “staircase steps” in the same bundles, i.e. the distance between different stereocilia rows, showed an over-growth of the majority of the 2nd and 3rd row stereocilia (Fig. 4d). In contrast to IHCs, neither row of OHC stereocilia in *Cib2* ^*tm1a/tm1a*^ mice exhibited elongated tips (Fig. 4c). To determine the effects of CIB2 deficiency on the known proteins responsible for stereocilia elongation, we explored localization of whirlin, myosin XVa, Eps8, and Eps8L2 in *Cib2* ^*tm1a/tm1a*^ mice. All four proteins were localized at the stereocilia tips in *Cib2* mutant mice (Supplementary Fig. 6b), suggesting that CIB2 is not essential for transporting them to the stereocilia tips. We concluded that, despite some differences between IHCs and OHCs, a common effect of CIB2 deficiency is the disruption of length regulation in the shorter row stereocilia and an over-elongation of the majority of them. It is worth mentioning that the shorter row stereocilia are the ones that harbor MET channels in mammalian auditory hair cells^3^, which may imply a link between CIB2 and mechanotransduction. We previously hypothesized this link based on the importance of CIB2 for hair cell function in zebrafish^19^.

### CIB2 is essential for auditory hair cell mechanotransduction

We investigated first whether the auditory hair cells of *Cib2* ^*tm1a/tm1a*^ mice have functional MET channels that are open at rest. We briefly incubated cultured organ of Corti explants from *Cib2* mutants with the MET channel-permeable dye FM1-43. In control explants, this incubation produced strong labeling of both OHCs and IHCs (Fig. 5a, Supplementary Fig. 7a), while no dye uptake was observed in *Cib2* ^*tm1a/tm1a*^ and *Cib2* ^*F91S/F91S*^ cochleae (Fig. 5b, Supplementary Fig. 7b). These data suggest that the MET channels in CIB2-deficient auditory hair cells are either closed at rest or non-functional.

Using conventional whole-cell patch-clamp recordings, we next studied MET currents in IHCs, because these cells have a less disrupted staircase bundle structure as compared to OHCs in *Cib2* mutants (Fig. 4a, b). Examination of IHC bundles with high-resolution SEM in freshly isolated organ of Corti explants revealed shorter but abundant links at the tips of 2nd and 3rd row stereocilia in *Cib2* ^*F91S/F91S*^ and *Cib2* ^*tm1a/tm1a*^ IHCs (Fig. 5c). We were not able to quantify the number of tip links in the *Cib2* ^*tm1a/tm1a*^ IHCs, due to very tight proximity between neighboring stereocilia in the bundle. However, we counted similar numbers of tip links in the IHCs of wild-type and *Cib2* ^*F91S/F91S*^ littermates (0.82 ± 0.03 links per stereocilium in wild-type vs. 0.84 ± 0.03 links in *Cib2* ^*F91S/F91S*^; number of cells, *n* = 12 and *n* = 10, correspondingly). After few days in culture, the tips of IHC 2nd and 3rd row stereocilia in *Cib2* mutants become more rounded and prominent tip links were observed (Supplementary Fig. 7c–e).

To deflect hair bundles, we used a fluid-jet, since it is able to produce not only positive but also very strong negative deflections of the bundle (Fig. 5d). Whole-cell patch-clamp recordings showed prominent MET currents in the control IHCs but not in *Cib2* ^*tm1a/tm1a*^ or *Cib2* ^*F91S/F91S*^ IHCs in both freshly isolated and cultured preparations (Fig. 5e, Supplementary Fig. 7c–e). In fact, we did not observe any MET current responses in *Cib2* mutants, even to the strongest bundle deflections that normally saturate MET current (Fig. 5f). In theory, MET current might be blocked by very large Ca^2+^ concentration inside stereocilia if, for some reason the intra-pipette Ca^2+^ buffer (1 mM of EGTA) was not strong enough to overcome the potential loss of Ca^2+^ buffering in *Cib2* mutants. Therefore, we recorded MET responses at large positive potentials that usually eliminate Ca^2+^ block to the MET channels^14, 17^. We did not detect any MET responses in all *Cib2* ^*F91S/F91S*^ IHCs (*n* = 6) that were tested at positive intracellular potentials (Fig. 5g). Complete loss of mechanotransduction in IHCs was observed in both strains of CIB2-deficient mice and in both acutely isolated (Fig. 5e–g) and cultured (Supplementary Fig. 7) preparations. We also did not observe any statistically significant differences in the resting concentration of free intracellular Ca^2+^ ([Ca^2+^]_i_) measured with Fura-2 indicator inside the OHCs of heterozygous and homozygous *Cib2* ^*tm1a/tm1a*^ mice (92.7 ± 1.6 nM, *n* = 15 and 93.0 ± 1.5 nM, *n* = 44, correspondingly, Mean ± SD, mid-cochlear location). Consistent with recent findings^30, 31^, IHCs at P4-P7 did not exhibit reverse-polarity mechanosensitive currents, i.e., the currents activated by negative bundle displacements, in both control and *Cib2-*mutant mice (Fig. 5e–g). However, these currents were observed in young OHCs of *Cib2* ^*tm1a/tm1a*^ mice (Fig. 5h, i), indicating that CIB2 deficiency does not affect Piezo2-associated^31^ reverse-polarity currents. In fact, the reverse polarity current seems to be accentuated by CIB2 deficiency and its developmental downregulation prolonged (Fig. 5i), exactly as observed in *Tmc1/Tmc2* double knockout mice^31^. We conclude that conventional mechanotransduction is impaired in *Cib2* ^*tm1a/tm1a*^ and *Cib2* ^*F91S/F91S*^ auditory hair cells.

### CIB2 interacts with TMC1 and TMC2

**Fig. 6:**
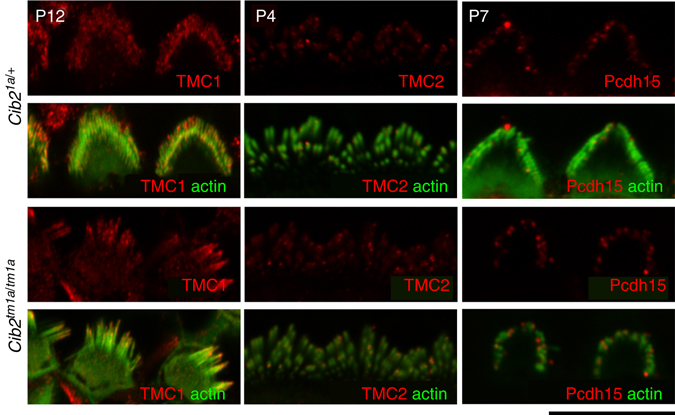
CIB2 deficiency does not mislocalize TMC1, TMC2 and Pcdh15 from the auditory hair cell stereocilia. Confocal images showing IHC stereocilia of *Cib2* ^*tm1a/+*^ and *Cib2* ^*tm1a/tm1a*^ mice immunostained with TMC1, TMC2, and Pcdh15 antibodies (*red*) and actin was labeled with phalloidin (*green*) at P12, P4 and P7 correspondingly. Scale bar: 10 μm

Because CIB2 is essential for mechanotransduction, we examined the localization of some known proteins associated with the tip links or the MET apparatus (PCDH15, TMC1, TMC2) in *Cib2* ^*tm1a/tm1a*^ mice using confocal imaging and found no differences from heterozygous control (Fig. 6), suggesting CIB2 is not essential for targeting of these proteins in stereocilia.

**Fig. 7:**
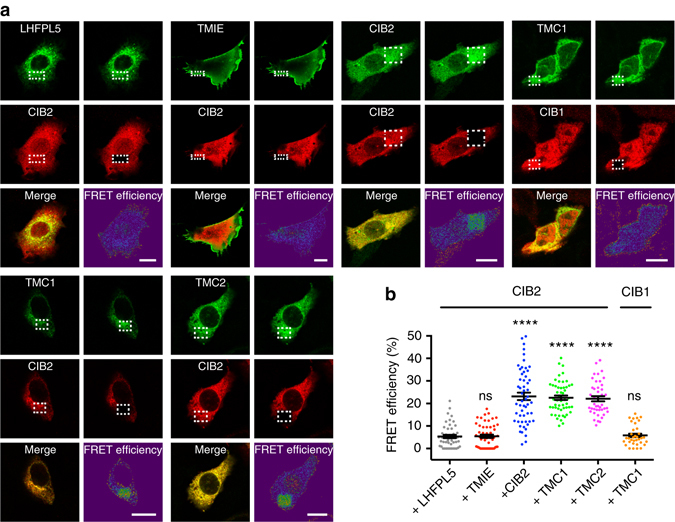
CIB2 interacts with TMC1 and TMC2. **a** Fluorescent images of cells co-expressing LHFPL5-GFP, TMIE-GFP, CIB2-GFP (positive control), TMC1-EGFP or TMC2-EGFP (donor) and CIB2-V5 (acceptor) before and after acceptor photobleaching within the indicated region (*white box*). For negative control, we used TMC1-EGFP (donor) and CIB1-V5 (acceptor). FRET efficiency images are calculated as *E*_FRET_ = 100 × (*I*_Post_−*I*_Pre_)/*I*_Post_, where *I*_Pre_ and *I*_Post_ are the EGFP pixel intensities before and after acceptor bleaching, respectively. Scale bars: 10 µm. **b** Quantitative analysis of FRET efficiency in CHO-K1 cells co-expressing LHFPL5-GFP, TMIE-GFP, CIB2-GFP, TMC1-EGFP or TMC2-EGFP (donor) and CIB2-V5 (acceptor). Quantitative analysis of negative control [TMC1-EGFP (donor) and CIB1-V5 (acceptor)] is also shown. *Asterisks* indicate statistical significance of FRET efficiency (*p* ≤ 0.0001). *Error bars* represent SEM. NS: not significant

Next, we examined the potential interaction of CIB2 with known components of the MET machinery including TMC1, TMC2, LHFPL5, and TMIE in heterologous cells using FRET assays. For positive control, we used FRET between CIB2 tagged with green fluorescent protein (GFP) and CIB2 tagged with V5, as our previous studies have shown that CIB2 can multimerize^19^. Although we cannot rule out the possibility of tags interfering with the interaction of CIB2 with TMIE and LHFPL5, we observed FRET interaction only between CIB2 and TMC1 and TMC2 (Fig. 7a, b), suggesting potential interactions between them. Concurrently, CIB2–TMC1 interaction was also identified in a yeast two-hybrid screening using N-terminus of TMC1 as a bait (Supplementary Fig. 8). These experiments also confirmed that CIB2 can form dimers. However, physiological significance of CIB2 multimerization is yet unclear.

**Fig. 8:**
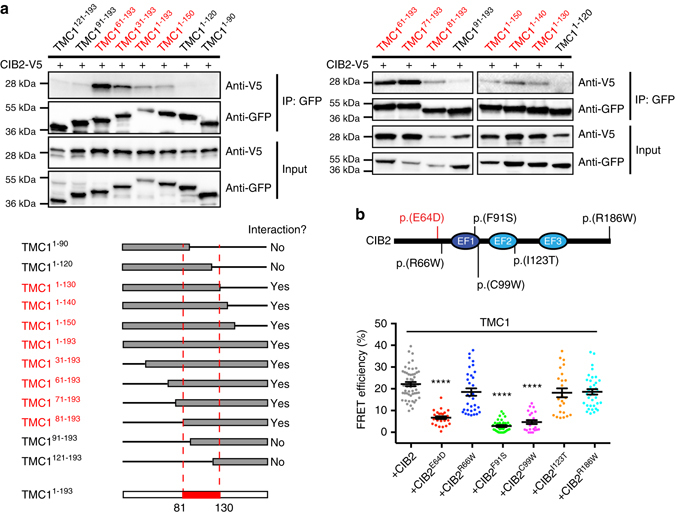
Molecular mechanisms of DFNB48/USH1J deafness. **a** Co-immunoprecipitation assays of differentially truncated N-terminal fragments of TMC1 and full-length CIB2 (*top panels*). Schematics of truncated N-terminal fragments of TMC1 are shown on the *bottom left panel*. Specific N-terminal domain of TMC1 (amino acids 81–130) is essential for binding to CIB2 (red dashed lines). **b** *Top*: The protein alteration identified in USH1J is shown in *red*, while the protein alterations identified in DFNB48 are shown in *black*. *Bottom*: Quantitative analysis of FRET efficiency in cells co-expressing EGFP tagged human TMC1 (donor) and V5 tagged human CIB2 with deafness causing mutations. *Error bars* represent SEM. *Asterisks* indicate statistical significance of FRET efficiency changes relative to the control interaction of TMC1 with wild-type CIB2 (ANOVA with Dunnett’s test)

To further confirm and map interaction domains, we generated deletion constructs of the N-terminus of TMC1 and tested their ability to associate with CIB2 in co-immunoprecipitation assays. Indeed, the N-terminus of TMC1, TMC1N (1–193), does interact and co-immunoprecipitate with CIB2 (Fig. 8a, *left*, Supplementary Figs. 8 and 11). Further deletion experiments demonstrated several other examples of TMC1-CIB2 interaction (Fig. 8a, *right*) and showed that a small domain (amino acids 81–130) in the TMC1 cytoplasmic N-terminus is critical for the TMC1-CIB2 interaction (Fig. 8a, *bottom left*). These results confirm that CIB2 is a binding partner of TMC1 and TMC2.

### Molecular mechanisms of DFNB48/USH1J deafness

We hypothesized that CIB2 may function as an accessary subunit essential for the MET channel activity. Therefore, the loss of hair cell mechanotransduction in *Cib2* mutants may result from the loss of TMC1-CIB2 interaction. If indeed CIB2 acts as an auxiliary subunit of MET channel complex, one would expect that CIB2 alleles, which do not affect targeting of CIB2 in stereocilia (e.g. p.F91S) might affect the interaction of CIB2 with TMC1, resulting in loss of MET current and hearing loss. To date, six *CIB2* missense mutations (Fig. 8b, *top*) causing Usher syndrome type 1 (p.E64D) and non-syndromic hearing loss (p.R66W, p.F91S, p.C99W, p.I123T, p.R186W) have been reported in humans^19, 29, 32^. None of these six missense mutations resulted in notable changes in CIB2 intracellular localization in CHO-K1 cells (Supplementary Fig. 9). We co-expressed the six mutants with human full length TMC1 and analyzed the effect of CIB2 mutations on association with TMC1 using FRET assays. Three out of six mutants (p.E64D, p.F91S, and p.C99W) affected interactions of CIB2 with TMC1 (Fig. 8b, *bottom*, Supplementary Fig. 10), suggesting that these mutations disrupt the putative TMC1-CIB2 complex in hair cells and thus cause deafness in humans.

## Discussion

This study provides the first evidence for the crucial role of CIB2 in both regulation of the lengths of transducing stereocilia and MET in mammalian auditory hair cells. However, the exact mechanisms of how CIB2 is involved in these processes require further investigation.

In *Cib2* mutant mice, the transducing shorter row stereocilia in the hair bundle are over-elongated in both OHCs and IHCs, while their tallest non-transducing stereocilia remain unaffected. Regulation of stereocilia lengths is likely to occur at their tips, which is the site of actin polymerization and remodeling^33, 34^. Several proteins involved in stereocilia length regulation were localized to the tips of transducing, shorter row stereocilia. The long isoform of myosin XVa is particularly interesting, because CIB2 may bind to whirlin at the tips of stereocilia^19^ and be a part of the myosin XVa/whirlin stereocilia elongation complex^35^. Alternatively, as a Ca^2+^ binding protein, CIB2 may buffer free Ca^2+^ in the vicinity of the MET channels at the tips of transducing stereocilia. It is known that changes in local Ca^2+^ concentration may initiate remodeling of actin cytoskeleton. An increase of Ca^2+^ initiates filopodia growth in cultured Helisoma neurons^36^. It has also been shown that increased Ca^2+^ concentration promotes the binding of actin-severing and capping proteins such as Villin^37^ and Gelsolin^38^, thereby reducing the level of actin polymerization. Therefore, it is possible that the potential decrease of Ca^2+^ buffering due to CIB2 deficiency will result in the overgrowth of transducing stereocilia observed in this study.

We found that early postnatal cochlear hair cells without functional CIB2 do not accumulate FM1-43 and have no detectable MET currents. These results suggest that CIB2 may either be essential for trafficking and assembly of the MET complex or be a component of this complex. Tip links remain intact in *Cib2* mutant mice, and essential proteins of the MET machinery (TMC1, TMC2, and PCDH15) are also properly localized in stereocilia. The lack of MET is observed even in *Cib2* ^*F91S/F91S*^ mice that have unchanged localization of the mutant CIB2 protein at the tips of stereocilia. Our ex vivo studies show that CIB2 binds to the cytoplasmic amino terminal of TMC1 and TMC2 proteins, and the p.F91S mutation disrupts this interaction. Mapping of TMC1-CIB2 interaction domains indicate that at least one distinct N-terminal domain of TMC1 is critical for CIB2 binding. Furthermore, not only N-terminal truncations of CIB2, but also several other deafness-associated missense mutations (p.E64D, p.F91S, and p.C99W), located in the central region of CIB2, also disrupt its interaction with TMC1. Finally, Piezo2-dependent reverse polarity MET currents in OHCs of *Cib2* mutants are still intact and similar to that in *Tmc1/Tmc2* double knockouts. Thus, we conclude that *Cib2* mutants may lack mechanotransduction because of the disruption of CIB2–TMC1/2 interactions. Perhaps, this interaction is essential for the normal function of TMC1 and TMC2. Although we did not observe interactions of CIB2 with other components of the transduction complex such as LHFPL5 and TMIE, we used epitope tagged molecules and, therefore, we cannot entirely dismiss this possibility, too.

As a Ca^2+^-sensitive component of the MET complex, CIB2 represents an ideal candidate for the proposed Ca^2+^ sensor of the intracellular “tension release” element responsible for fast adaptation of the MET responses in mammalian and non-mammalian hair cells^39–41^. Ca^2+^ has been shown to bind to CIB2 and to induce a conformational change in the protein with exposure of hydrophobic residues^27^. Depending on the exact structure of the CIB2–TMC1 complex, which is yet unknown, CIB2 changes may alter the tension within the MET complex and cause adaptation either directly or by initiating further changes in this complex. Since we were not able to detect any MET current in the cochlear hair cells of *Cib2* mutants, we cannot, unfortunately, prove or disprove this hypothesis. Perhaps other deafness-related mutations (e.g. p.R186W) in *Cib2* might be less deleterious to hair cell mechanotransduction and might clarify the role of CIB2 in the adaptation of MET current when corresponding *Cib2* mouse knockin models become available. Considering the known interactions with whirlin and myosin VIIa^19^, CIB2 is still a good candidate for the proposed Ca^2+^-sensitive “tension release” element ^39, 40^ linking the MET complex at the plasma membrane and the cytoskeletal elements at the tips of the transducing stereocilia.

So far, six missense mutations of *CIB2* have been reported as responsible for hearing loss in humans. Three of these mutations (p.E64D, p.R66W, and p.F91S) are present in and around the first EF hand domain (Fig. 8b) and our in vitro Ca^2+^ imaging suggest that these mutations do not significantly affect the Ca^2+^ buffering ability of CIB2^19, 29^^.^ Interestingly, in this study we show that two of these mutations (p.E64D and p.F91S) affect the interaction of CIB2 with TMC1 and TMC2. At least one of them, p.F91S leads to the loss of MET currents, as observed in the *Cib2* ^*F91S/F91S*^ knock-in mice. The p.C99W mutation is located within the interface of the effector-binding (EF1) and Ca^2+^-binding (EF2) domains and affects both the interaction with TMC1 (Fig. 8b) and Ca^2+^ buffering abilities of CIB2^19^. In contrast, the p.I123T and p.R186W mutations only impair the Ca^2+^ buffering of CIB2^19, 32^ and thus are excellent candidates to explore the potential role of CIB2 in maintaining Ca^2+^ homeostasis in the auditory hair cells. In conclusion, our study demonstrates that CIB2 is required for normal operation of the mechanotransducer channels and for hearing in mice. Notably, IHCs remain intact for a few postnatal weeks in *Cib2* mutants, which might provide a therapeutic window for the treatment of *Cib2*-related hearing impairments.

## Methods

### *Cib2* mutant mouse models

*Cib2* ^*tm1a*^ mice: *Cib2* ^*tm1a(EUCOMM)Wtsi*^ mice were obtained from the EUCOMM repository and maintained on C57BL/6 N background. A gene trap cassette containing lacZ and neomycin resistance genes flanked by FRT sites was inserted downstream of exon 3, with exon 4 flanked by loxP sites (Fig. 1a).

*Cib2* ^*tm1b*^ mice: As part of the International Mouse Phenotyping Consortium (IMPC) program^42^, *Cib2* ^*tm1b(EUCOMM)Wtsi*^ mutant mice (*Cib2* ^*tm1b*^) were produced, which like the *Cib2* ^*tm1a*^ allele, retain a gene trap in intron 3, but have had the neomycin selection cassette and exon 4 removed by Cre–LoxP recombination (Fig. 1a).

*Cib2* ^*F91S/F91S*^ mice: To generate *Cib2* ^*F91S/F91S*^ knock-in mice (*Cib2* ^*F91S/F91S*^), a 6.65-kb region, including *Cib2* exon 4 was amplified from the genomic DNA of 129X1/SvJ mice, and c.272 T > C mutation (resulting in p.F91S missense allele in the encoded protein) was introduced through site-directed mutagenesis. For ES cells selection, a neomycin resistance cassette that was flanked by FRT sites was also included in the targeting construct. Southern blot analysis was performed to identify the correctly targeted ES cells. Three of the correctly targeted ES cells were used for injection into C57BL/6 J blastocysts, and resulting chimeric mice were mated with C57BL/6 N females to generate heterozygous mice. The resulting *Cib2* ^*F91S*^ mouse strain was maintained on a C57BL/6 N background. For the data analysis, the data from males and females were mixed together. The age of the mice used for experiments is indicated in each figure legend. All experiments and procedures were approved by the Institutional Animal Care and Use Committees of the University of Maryland School of Medicine, Baltimore, and the University of Kentucky, Lexington.

### ABR and DPOAE measurements

Hearing thresholds of *Cib2* ^*tm1a*^ and *Cib2* ^*F91S*^ mice at P16 (*n* = 5–10 each genotype) were evaluated by recording ABR and DPOAE. All ABR recordings, including broadband clicks and tone-burst stimuli at three frequencies (8, 16, and 32 kHz), were performed using an auditory-evoked potential RZ6-based auditory workstation (Tucker-Davis Technologies) with high frequency transducer. Maximum sound intensity tested was 100 dB SPL. TDT system III hardware and BioSigRZ software (Tucker Davis Technology) were used for stimulus presentation and response averaging. DPOAEs were recorded at P16 (*n* = 5–10) from wild-type, heterozygous and *Cib2* ^*tm1a*^ and *Cib2* ^*F91S*^ homozygous mice using an acoustic probe (ER-10C, Etymotic Research) and DP2000 system (version 3.0, Starkey Laboratory). To measure DPOAE levels (2f1-f2), two primary tones, with a frequency ratio of f2/f1 = 1.2, were presented at intensity levels f1 = 65 dB SPL and f2 = 55 dB SPL, with f2 varied between 8–16 kHz (in one-eighth octave steps).

### X-Gal staining

Activity of the *lacZ* reporter gene product, β-galactosidase, was detected by X-gal staining in the inner ear. For whole mount X-gal staining, temporal bones were fixed for 20 min with 1% formaldehyde, 0.2% glutaraldehyde, and 0.02% NP-40 diluted in phosphate-buffered saline (PBS), followed by washing for 1 h with 1× PBS containing 0.02% NP-40 and 2 mM MgCl_2_. Temporal bones were stained for 3 h at 37 °C in the dark with X-gal solution [1 mg/ml X-gal, 5 mM K3Fe(CN)6, 5 mM K4Fe(CN)6, and 0.1 M MgCl_2_ in PBS]. After washing, temporal bones were incubated overnight in 0.25 M EDTA and 2% para formaldehyde (PFA) solution. Inner ears were fine dissected and imaged using a system (AxioVision Z1; Carl Zeiss, Inc.) fitted with an Apotome slide module with a 63× NA 1.4 Plan-Apochromatic objective and a digital camera (AxioCam MRm; Carl Zeiss, Inc.).

Whole-mount embryonic day 12.5 (E12.5) embryos and adult X-gal stainings of *Cib2* ^*tm1b/+*^ animals were performed as previously described^43^. Embryos were fixed for 20 min in cold 4% PFA in PBS (pH 8.0), followed by washing, and stained for 48 h at 4 °C in the dark under gentle agitation. Adult mice were anaesthetized and trans-cardially perfused with 4% PFA in PBS (pH 8.0). Blocks of tissues were further fixed for 30 min, stained with standard X-gal staining solution at 4 °C for 48 h in the dark under gentle agitation, post-fixed, and cleared with 50% glycerol/PBS followed by 70% glycerol/PBS. After post-fixation, embryos and adult organs were dissected out and placed on Sylgard lined petri dishes and imaged using a LeicaM165C stereomicroscope with a 0.63 objective Planapo and a Jenoptik Prog Res Speed XT core 5 camera.

### Antibodies

The following primary commercially available antibodies were used: anti-KDEL (1:500, Life Technologies #PA1-013), anti-V5 (1:500, Life Technologies #R960-25), anti-GFP (1:1000, ChromoTek #029762), anti-CIB2 (1:200, Abnova #H00010518-A01), anti-myosin VIIa (1:300, Proteus Biosciences #25-6790), anti-TMC1 (1:50, Sigma #HPA 044166), anti-Eps8 (1:50, BD Biosciences #610144) and anti-β-galactosidase (1:500, MP #559761). The following rabbit polyclonal, affinity purified primary custom made antibodies were obtained for the studies: anti-TMC2 (1:200, PB361)^44^, anti-whirlin (1:200, PB5140)^35^, anti-myosin XVa (1:50, PB48)^45^, anti-Eps8L2 (1:50, NP_073609.2)^46^ and anti-Pcdh15 (1:200, HL5614)^5^. Secondary antibodies were: Alexa Fluor-405, 488, 546, or 647 goat anti-rabbit and Alexa Fluor-546, 555, or 647 goat anti-mouse (1:1000, Life Technologies).

### Immunostaining and confocal imaging

24 h after transfection, CHO-K1 cells were plated on chambered coverslips (Nunc Lab-Tek II) in Ham’s F12 supplemented with 10% fetal bovine serum (FBS) at 37 °C for 24 h. Before staining, the samples were washed twice with PBS, fixed with 4% PFA (15 min, 37 °C), washed three times with PBS, and permeabilized and blocked with 5% normal serum and 0.25% Triton X-100 in PBS (60 min, room temperature). Then, the samples were incubated with primary antibodies (overnight, 4 °C), washed five times with PBS, and incubated with secondary antibodies for 2 h at room temperature.

Temporal bones were fixed and processed for immunocytochemistry as described previously^19^. For TMC1 immunostaining, temporal bones were processed as described previously^47^. The cochlear and vestibular sensory epithelia were isolated, fine dissected and permeabilized in 0.25% Triton X-100 for 1 h, and blocked with 10% normal goat serum in PBS for 1 h. The tissue samples were probed with primary antibody overnight and after three washes were incubated with the secondary antibody for 45 min at room temperature. Rhodamine phalloidin or phalloidin 647 (Invitrogen) was used at a 1:250 dilution for F-actin labeling. Nuclei were stained with DAPI (Molecular Probes). All images were acquired using a LSM 700 laser scanning confocal microscope (Zeiss, Germany) using a 63× 1.4 NA or 100× 1.4 NA oil immersion objectives. Stacks of confocal images were acquired with a Z step of 0.5 µm and processed using ImageJ software (National Institutes of Health). Experiments were repeated at least 3 times, using at least three different animals.

### Paraffin sectioning and hematoxylin and eosin staining

Temporal bones from P60 *Cib2* mutant mice were harvested and fixed in 4% PFA solution for 24 h at 4 °C. Inner ears were then decalcified using 0.25 M EDTA for 2 days and after washing with 1× PBS processed for paraffin embedding. Paraffin blocks were sectioned at 6 µm thickness, stained with hematoxylin and eosin and mounted with xylene.

### Scanning electron microscopy

*Cib2* mutant and control cochleae were fixed in 2.5% glutaraldehyde in 0.1 M cacodylate buffer supplemented with 2 mM CaCl_2_ for 1–2 h at room temperature. Then, the sensory epithelia were dissected in distilled water. Specimens were dehydrated in a graded series of ethanol, dried at the critical point from liquid CO_2_, sputter coated with platinum (5.0 nm, controlled by a film-thickness monitor), and observed with a field-emission SEM (Hitachi S-4300, S-4800, or SU-8010).

### Acceptor photobleaching FRET

Transfection, plating, fixation and staining of cells, and mounting of samples were performed as described previously^19^. Acceptor photobleaching FRET analysis was performed using a Leica TCS SP8 confocal laser scanning microscope and FRET-AB wizard of LEICA LAS AF software. Cells expressing various EGFP and V5 tag fusions were incubated with monoclonal antibody specific to V5 tag overnight at 4 °C. Anti-V5 antibody was subsequently labeled with highly cross-adsorbed goat anti-mouse Alexa Fluor 555. We chose EGFP and Alexa Fluor 555 dye as the donor–acceptor pair because the R0 value for FRET between EGFP and Alexa Fluor 555 is 63 Å (R0 value in angstroms (Å) represents the distance at which FRET from the donor dye to the acceptor dye is 50% efficient). We performed photobleaching with 100% 561 nm laser power for the acceptor. EGFP donor fluorescence was imaged before and after bleaching a region of interest (ROI) of Alexa Fluor 555 to less than 10% of its initial intensity. FRET efficiency was calculated as *E*_FRET_ = 100 × (*I*_Post_—*I*_Pre_)/*I*_Post_, where *I*_Pre_ and *I*_Post_ stand for the EGFP intensities before and after acceptor bleaching, respectively.

### FM1-43 dye uptake experiment

Cochlear and vestibular explants from *Cib2* mutant and control mice were dissected at postnatal day 5 (P5) and cultured in a glass-bottom petri dish (MatTek, Ashland, MA). They were maintained in Dulbecco's modified Eagle medium (DMEM) supplemented with 10% FBS (Life Technologies) for 2 days at 37 °C and 5% CO_2_. Explants were incubated for 10 s with 3 µM FM1-43, washed three times with Hank's balanced salt solution, and imaged live using a Zeiss LSM 700 scanning confocal microscope.

### Co-Immunoprecipitation assay

HEK 293 cells were maintained at 37 °C in 5% CO_2_ using DMEM supplemented with 10% FBS, glutamine and penicillin–streptomycin (Invitrogen). Cells grown in 100-mm culture dish were transfected using 10 μg of each cDNA using polyethylenimine (Polysciences). Forty-eight hours post transfection, cells were washed with fresh 1× PBS, and sonicated (Fisher Scientific) for 10 s in RIPA buffer containing a protease inhibitor mixture (Roche). Protein A–Sepharose CL-4B beads were incubated for 4 h with 5 μg of GFP antibody, followed by washing three times with cold PBS containing 0.1% Triton X-100. Cell lysates were incubated with GFP antibody coated CL-4B beads overnight at 4 °C. The following day, beads were centrifuged at 10,000 *g* for 3 min, washed three times with RIPA buffer, and boiled for 5 min in 2× SDS sample buffer. Samples were electrophoresed and processed for western blot using 4–20% Tris-Glycine gel (Novex). Images have been cropped for presentation. Full size images are presented in Supplementary Fig. 11.

### Whole-cell patch-clamp recording

MET responses were recorded using the conventional whole-cell patch-clamp technique as described previously^48^. Experiments were performed at room temperature in Leibovitz (L-15) cell culture medium (Invitrogen) containing the following inorganic salts: 137 mM NaCl, 5.4 mM KCl, 1.26 mM CaCl_2_, 1 mM MgCl_2_, 1 mM Na_2_HPO_4_, 0.44 mM KH_2_PO_4_, and 0.81 mM MgSO_4_. We used either cultured organ of Corti explants harvested at P4-P5 and kept in vitro for 1–3 days or acutely isolated organs of Corti at P1-P10.

To access the basolateral surface of IHCs, we either removed the neighboring cells with a gentle suction (in cultured preparations), or approached IHCs in the acutely isolated samples with a large positive pressure in the patch pipette, which separates the IHCs from the rest of the tissue. Patch-clamp pipettes were filled with: 140 mM CsCl, 2.5 mM MgCl_2_, 2.5 mM Na_2_ATP, 1 mM EGTA, and 5 mM HEPES, osmolarity 325 mOsm, pH = 7.35. The pipette resistance was typically ~ 5 MΩ. Series resistance was not compensated, because relatively slow hair bundle deflections with a fluid-jet (~1 ms rise time) resulted in a rather slow rise of MET current, which did not require a fast voltage clamp. The uncompensated voltage drops across the access resistance did not exceed 7.5 mV. IHCs were held at a potential of −60 mV between the recordings and at −90 mV for the transient periods of MET recordings, unless stated otherwise.

IHC bundles were deflected with a fluid-jet^49^. Pressure was generated using a high speed pressure clamp (HSPC-1, ALA Scientific) and applied to the back of a ~5 μm pipette filled with the bath solution. The pipette tip was positioned at a distance of ~8 μm in front of the hair bundle. It was determined that the force generated by this microjet depends linearly on the applied pressure^49^. Before each experiment, the steady-state pressure was adjusted to zero by monitoring debris movement in front of a fluid-jet. All recordings were made from the IHCs in the apical cochlear turn.

To record reverse-polarity currents in OHCs, hair bundles were deflected with a fluid jet, driven by a 40-Hz sinusoidal voltage applied to a 27-mm-diameter piezoelectric disc to produce fluid jet and elicit maximal MET current. Whole-cell recordings were made with patch electrodes filled with: 130 mM CsCl, 3 mM MgATP, 10 mM Tris phosphocreatine, 1 mM EGTA, 10 mM Cs-Hepes, pH 7.2, ∼295 mOsm/l, and connected to an Axopatch 200 A amplifier with 5-kHz output filter. OHCs were clamped at −70 mV. All recordings were made from apical OHCs.

The hair bundle motion elicited by the fluid jet was calibrated in some experiments by imaging the bundle on a pair of photodiodes^50^. The calibration factors were very reproducible between experiments, with the values of 4 nm/V and 6 nm/mm Hg for the piezoelectric disk-driven fluid jet stimulating OHCs and pressure clamp-driven fluid jet stimulating IHCs, correspondingly.

### RT-qPCR

RNAs from cochlea and vestibular system were extracted at P12 from *Cib2* heterogygous and homozygous mice using the Ribopure kit (Ambion). SMARTScribe Reverse Transcriptase kit (Clontech) was used to generate cDNAs, and SYBRgreen technology (Qiagen) was used to perform the qPCR. The following primers were used to amplify Cib1-4: mCIB1_F: 5ʹ-tggaaccctggacagagaa-3ʹ; mCIB1_R: 5ʹ-ccctgtcaatgtctgactcttc-3ʹ; mCIB2_F: 5ʹ-ctctgtgctctgcgaatca-3ʹ; mCIB2_R: 5ʹ-ccagcgtcatctctaagtctt-3ʹ; mCIB3_F: 5ʹ-ggatggtcacatgaccttagag-3ʹ; mCIB3_R: 5ʹ-gtcccatgcacagatgtagtt-3ʹ; mCIB4_F: 5ʹ-tggtttcattgatgaggagga-3ʹ; mCIB4_R: 5ʹ-agatctgactcactcaggacat-3ʹ. *Gapdh* amplification was used to normalize the samples: mGAPDH_F: 5ʹ-tcaacagcaactcccactcttcca-3ʹ; mGAPDH_R: 5ʹ-accctgttgctgtagccgtattca-3ʹ.

### Yeast two-hybrid assay

The coding sequence of mouse Tmc1 (aa.1–193, GenBank accession number gi: 119703759) was PCR-amplified and cloned in frame with the Gal4 DNA binding domain into plasmid pB66 (N-GAL4-Tmc1-C fusion), derived from the original pAS2ΔΔ vector. The prey fragment for mouse full length Cib2 (GenBank accession number gi: 148747105) was cloned in frame with the Gal4 activation domain into plasmid pP6, derived from the original pGADGH vector. The pP7 prey plasmid used in the control assay is derived from the pP6 plasmid.

Baits and preys constructs were transformed in the yeast haploid cells, respectively L40∆Gal4 (mata) and YHGX13 (Y187 ade2-101::loxP-kanMX-loxP, matα) strains. The diploid yeast cells were obtained using a mating protocol with both yeast strains. These assays are based on the HIS3 reporter gene (growth assay without histidine). Interaction pairs were tested in duplicate as two independent clones from each diploid were picked for the growth assay. For each interaction, the diploid yeast cells expressing both bait and prey constructs were spotted on several selective media. The selective medium lacking tryptophan and leucine was used as a growth control and to verify the presence of both the bait and prey plasmids. The diploid yeast cells were also spotted on a selective medium without tryptophan, leucine and histidine.

### Statistical analyses

Immuno samples were assayed in triplicate. For in vitro analyses, each experiment was repeated at least three times. All error bars represent SEM. One way ANOVA with Dunnett’s test was used to compare the different groups of independent samples. The level of statistical significance was set at 0.05 for all tests.

### Data availability

The data supporting the findings of this study are available from the corresponding authors upon request.

## Electronic supplementary material

Supplementary Information
