# Supplementary material for: CIB2 interacts with TMC1 and TMC2 and is essential for mechanotransduction in auditory hair cells
Source: Nat Commun. 2017 Jun 29;8:43. doi: 10.1038/s41467-017-00061-1 (PMC5491523; doi:10.1038/s41467-017-00061-1)
Supplement: Supplementary file 1 — Supplementary Information [file 41467_2017_61_MOESM1_ESM.pdf]

File name: Supplementary Information
Description: Supplementary Figures and Supplementary Table

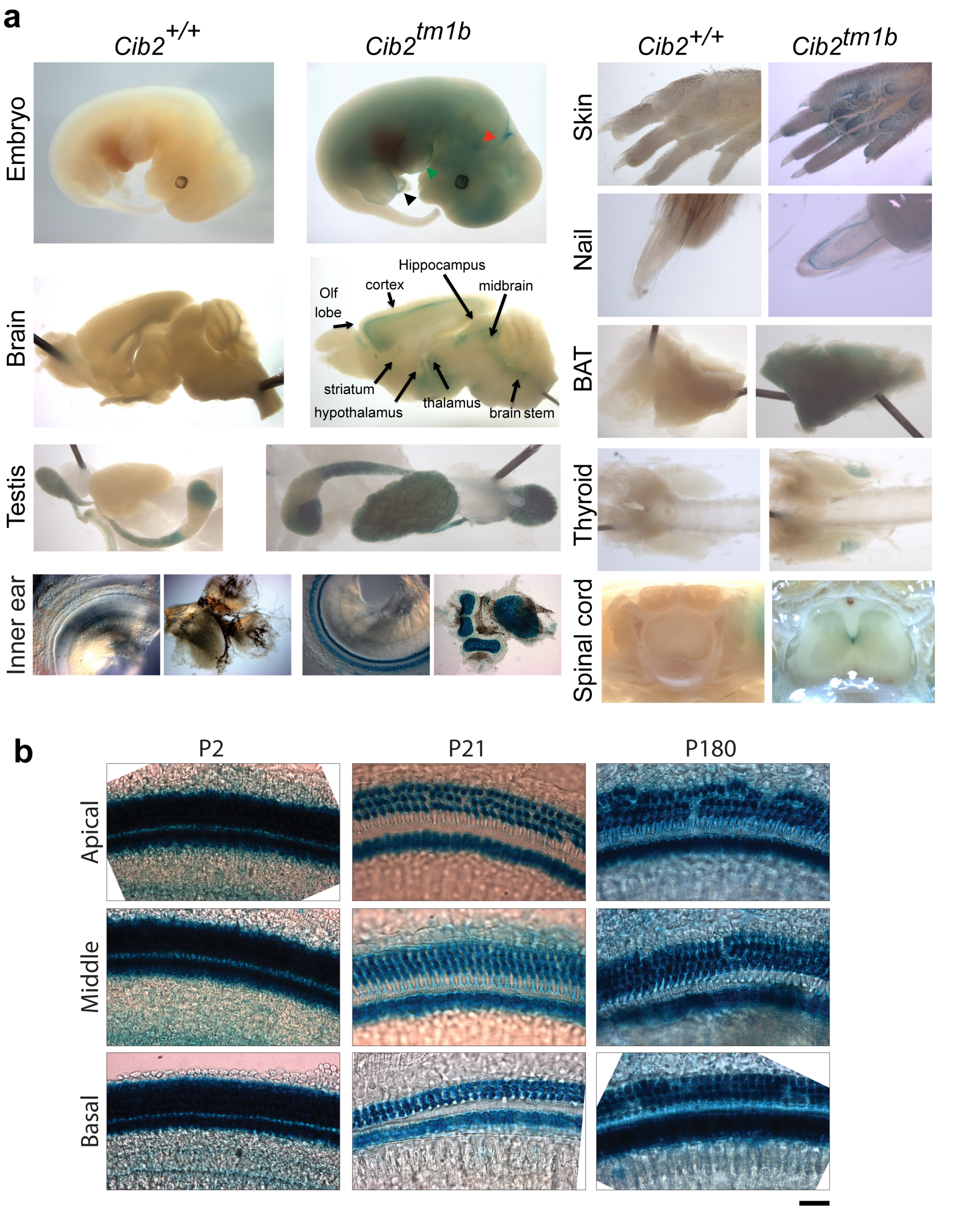

Supplementary Fig. 1 β-galactosidase activity, recapitulating the transcriptional activity of the *Cib2* locus *in Cib2*$^{+/+}$ and *Cib2*^{tm1b} mice

(**a**) *Cib2* is ubiquitously expressed and is observed in many locations, including the brain, the testis, the cochlea, the vestibular organs, the skin, the nails, the thyroid, the brown

adipose tissue (BAT) and the spinal cord. In *Cib2*$^{tm1b/+}$ E12.5 embryos, reporter expression is observed in the inner ear (red arrowhead), the apical ectodermal ridge (green arrowhead), and the umbilical cord (black arrowhead). Transcriptional activity is also recorded in adult *Cib2*$^{tm1b/+}$ mice. (**b**) X-Gal staining showing the expression of *Cib2* in the apical, medial and basal region of the organ of Corti of *Cib2*$^{tm1a/+}$ mice at postnatal day 2 (P2), P21 and P180. At P2, *Cib2* is expressed in both sensory hair cells and supporting cells of the organ of Corti. Later in development, the expression of *Cib2* is more pronounced in the sensory hair cells of the organ of Corti. Scale bar is 20 μm.

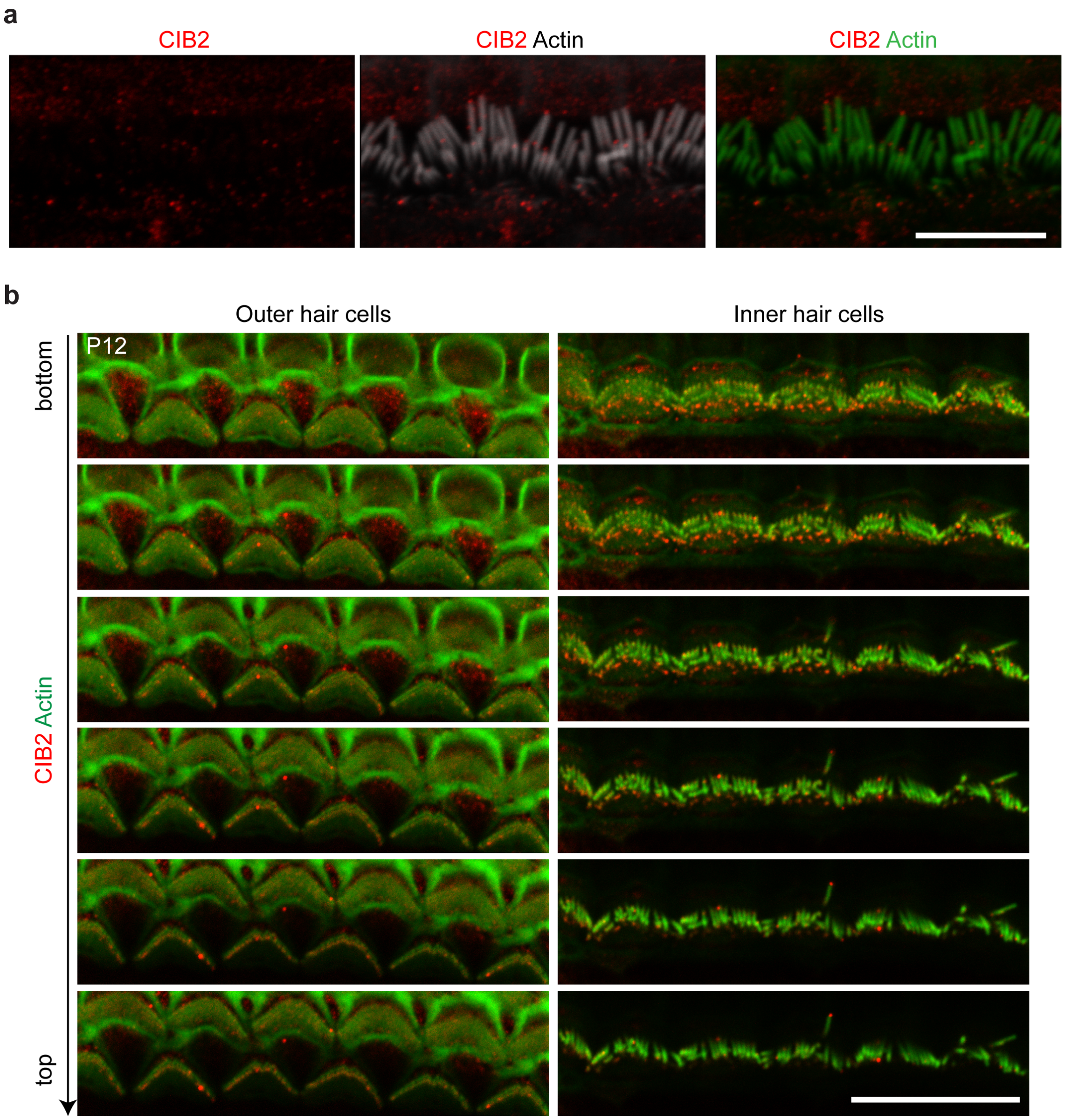

Supplementary Fig. 2 CIB2 is localized in the stereocilia of auditory hair cells

(**a**) Confocal images of P5 control IHCs immunostained with CIB2 antibody (red) and counterstained with phalloidin (green) (**b**) Confocal images of P12 wild-type OHCs and IHCs immunostained with CIB2 antibody (red) and counterstained with phalloidin (green). The six traces are confocal sections from the *bottom* to the *top* of the bundles, z step. Scale bars: (**a**) 5µm, (**b**) 10µm and z is 0.5µm.

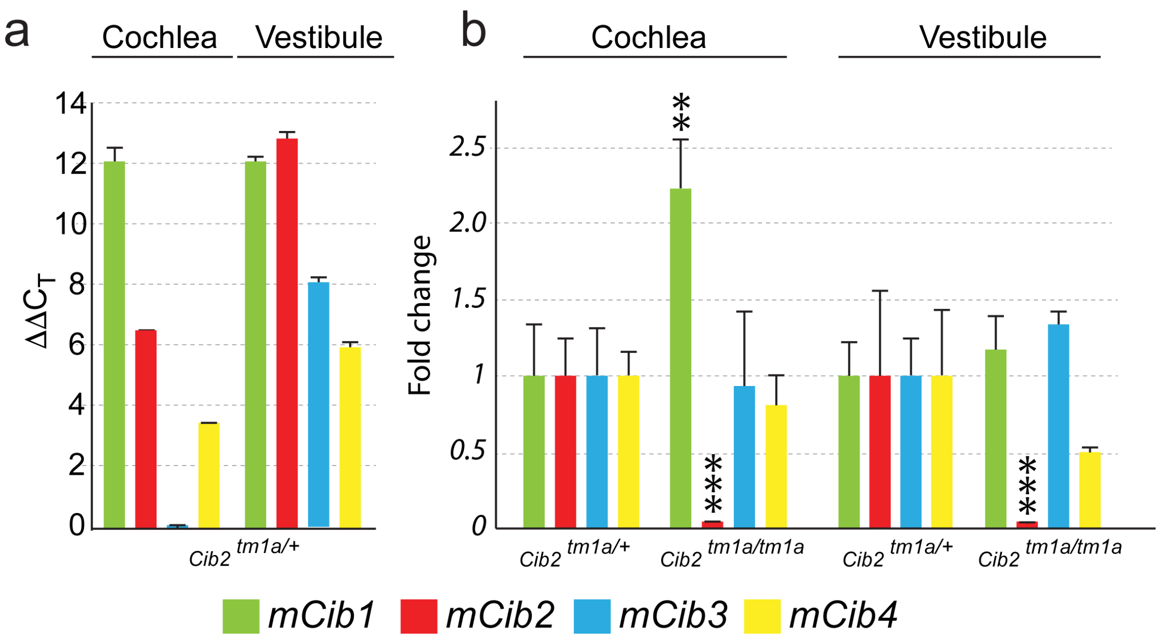

Supplementary Fig. 3 Expression of *Cib* genes in mouse cochlear and vestibular end organs

Real-time quantitative RT–PCR analysis of mRNA levels in the cochlear and vestibular sensory epithelia at P12 revealed different expression of *Cib* family members in the auditory and vestibular end organs (**a**) and significant ($p<0.001$) compensatory overexpression of *Cib1* in the cochlea of $Cib2^{tm1a/tm1a}$ mice (**b**). (**a**) The relative expression of *Cib* family members in the cochlear and vestibular samples of $Cib2^{tm1a/+}$ mice were normalized against *Cib3* expression in the cochlea. (**b**) The relative expressions of *Cib* genes in $Cib2^{tm1a/tm1a}$ mice were normalized against their corresponding expressions in $Cib2^{tm1a/+}$ mice. Asterisks indicate statistical significance: **, $p<0.01$; ***, $p<0.001$ (Student's *t*-test). Error bars represent SEM.

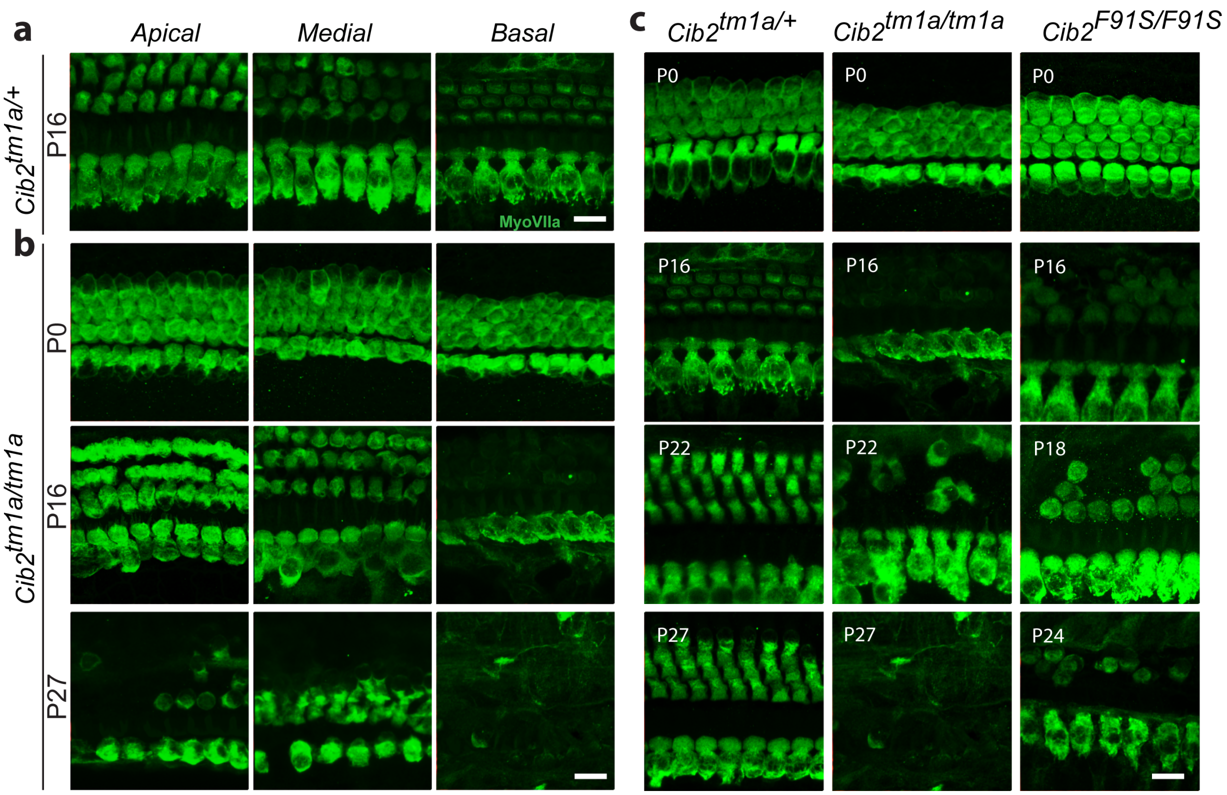

Supplementary Fig. 4 *Cib2* mutations lead to degeneration of the organ of Corti

(**a-b**) Maximum intensity projections of confocal Z-stacks of the apical (left), medial (middle), and basal (right) turns of *Cib2*$^{tm1a/+}$ (**a**) and *Cib2*$^{tm1a/tm1a}$ (**b**) organs of Corti immunostained with myosin VIIa antibody (green) at different postnatal ages (P0-P27) indicated on the panels. (**c**) Maximum intensity projections of confocal Z-stacks of the medial/basal turns of *Cib2*$^{tm1a/+}$ (right), *Cib2*$^{tm1a/tm1a}$ (middle) and *Cib2*$^{F91S/F91S}$ (left) organs of Corti immunostained with myosin VIIa antibody (green) at different postnatal ages (P0-P27) indicated on the panels. Scale bars: 10 µm.

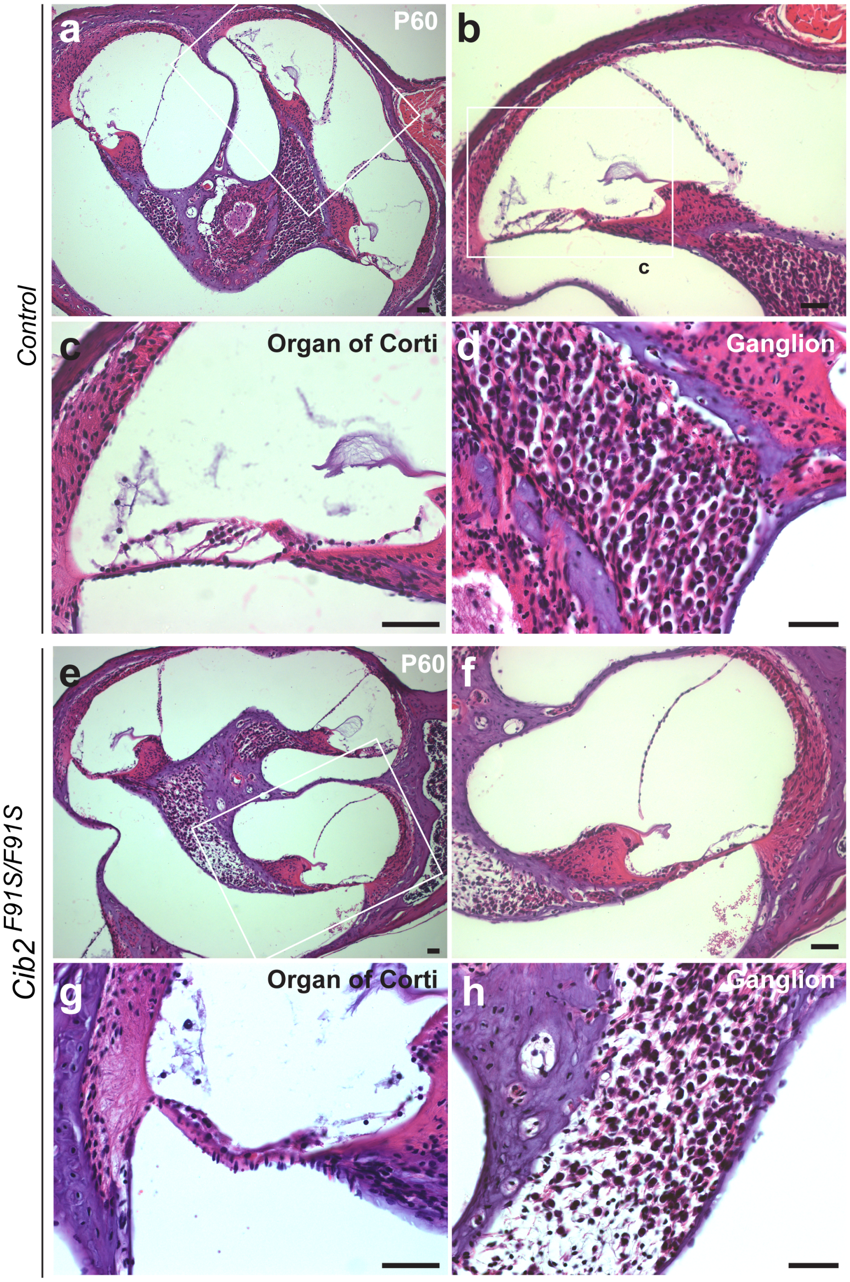

Supplementary Fig. 5 The spiral ganglion neurons in adult *Cib2* mutant mice progressively degenerate

(**a**-**h**) Hematoxylin and eosin staining of paraffin section of P60 inner ear of control (**a**-**d**) and homozygous *Cib2*^{F91S} mice (**e**-**h**) revealed degeneration of spiral ganglion neurons. Boxed regions are magnified for close-up view of the organ of Corti and spiral ganglion cells. Scale bars: 100μm.

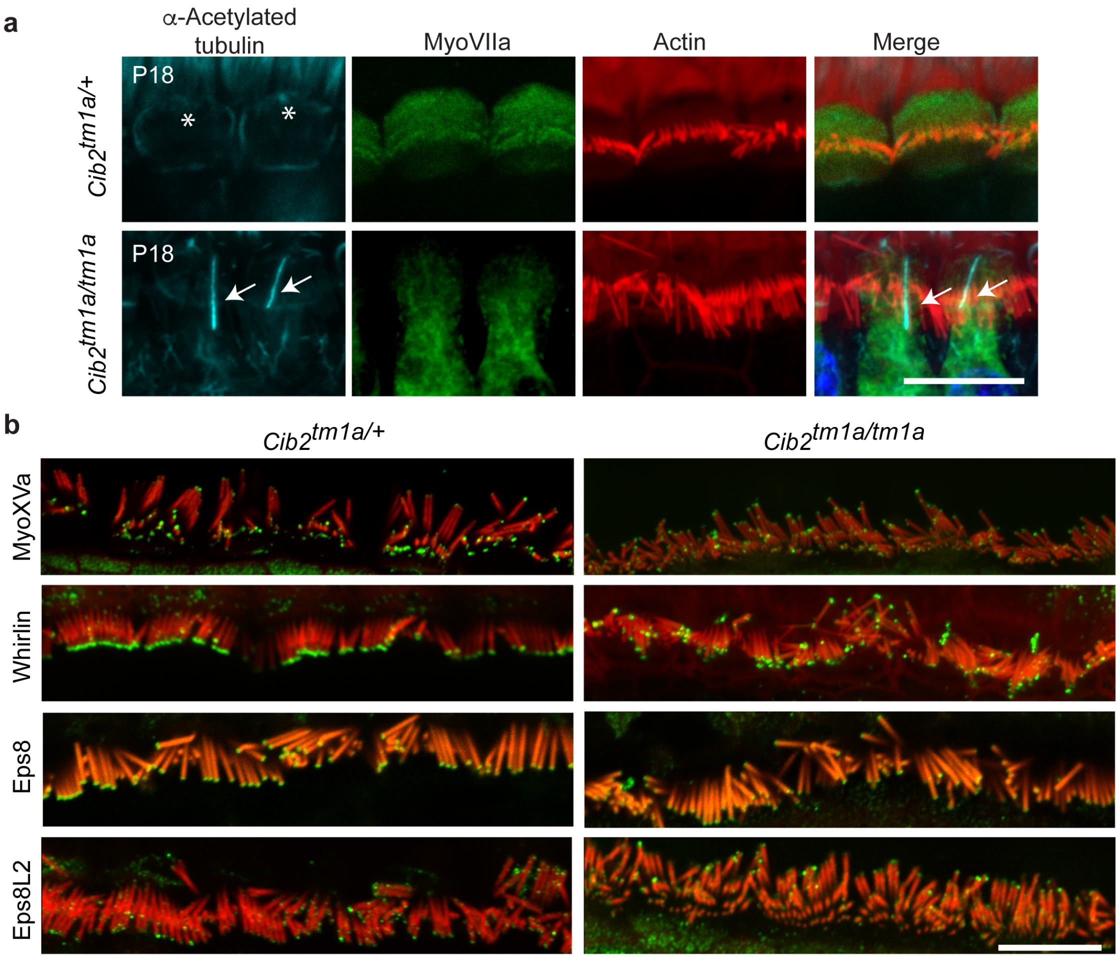

Supplementary Fig. 6 Defects in the staircase architecture of stereocilia bundles of *Cib2* mutants occur without mislocalization of the proteins involved in the stereocilia elongation

(**a**) Confocal images showing immunolabeling of the kinocilium with α-acetylated-tubulin (cyan, arrows) in the auditory hair cells (MyoVIIa, green) of P18 *Cib2*$^{tm1a/+}$ and *Cib2*$^{tm1a/tm1a}$ mice. Phalloidin staining is shown in red; DAPI staining is shown in blue. The asterisks are showing the absence of kinocilia in control mice. (**b**) Confocal images of the middle turns of P12 *Cib2*$^{tm1a/+}$ (left) and *Cib2*$^{tm1a/tm1a}$ (right) organs of Corti immunostained with myosin XVa, whirlin, Eps8, and Eps8L2 antibodies (all in green) and counterstained with phalloidin (red). Scale bars: 10 μm.

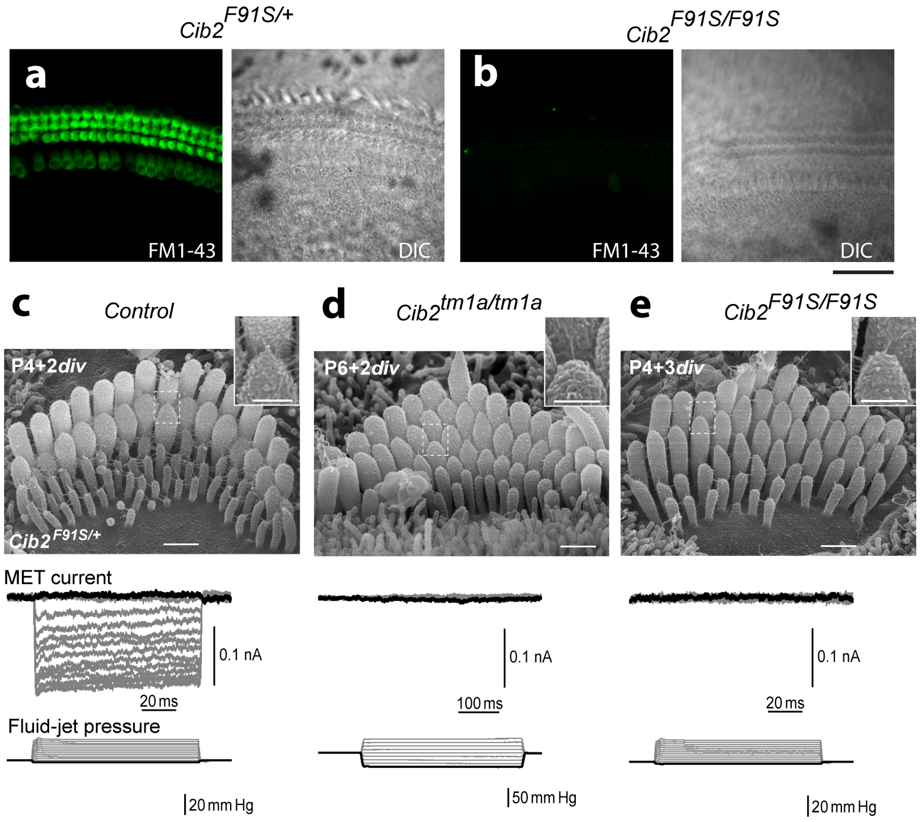

Supplementary Fig. 7 CIB2 is essential for hair cell mechanotransduction

(**a**, **b**) Maximum intensity projections of Z-stacks of confocal fluorescent images (left) and corresponding DIC images (right) of control (**a**) and *Cib2*$^{F91S/F91S}$ (**b**) cultured organ of Corti explants imaged after exposure to 3µM of FM1-43 for 10s. The samples were dissected at P5 and kept two days in vitro (P5+2*div*). Scale bar: 20 µm. (**c-e)** SEM images of IHCs (top panels) in cultured organ of Corti explants from control (**c**), *Cib2*$^{tm1a/tm1a}$ (**d**), and *Cib2*$^{F91S/F91S}$ (**e**) mice and MET currents evoked by fluid-jet deflection of stereocilia (bottom) in the same explants that are illustrated on top SEM micrograph. The black traces show the responses to the strongest negative deflections of the bundle. The insets at the top panels show the magnified images of tip links. Ages of the cells are indicated in the top left corners of the SEM images. Scale bars in panels c, d and e are 0.5 µm. Scale bars in inserts are 200 nm.

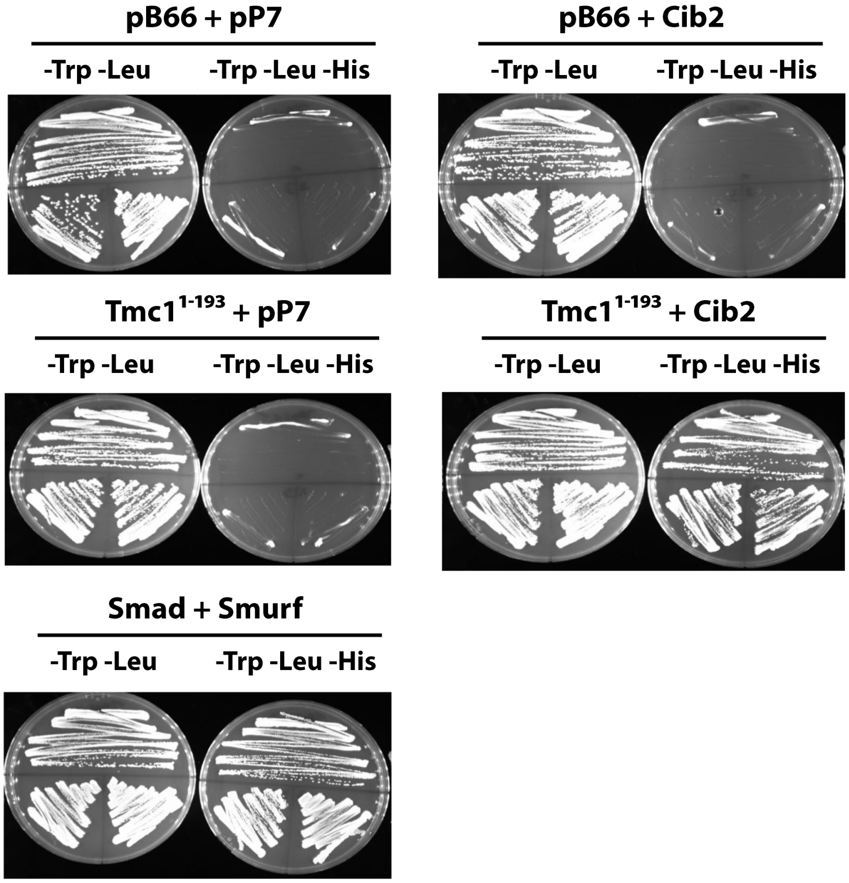

Supplementary Fig. 8 The N-terminus of mouse TMC1 (1-193 aa) can interact with CIB2

Interaction pairs were tested in triplicate as three independent clones from each diploid were picked for the growth assay. For each interaction, the diploid yeast cells expressing both bait and prey constructs were spotted on several selective media. The selective medium lacking tryptophan and leucine was used as a growth control and to verify the presence of both the bait and prey plasmids (the left plate in each panel). Protein-protein interactions were detected on a selective medium without tryptophan, leucine, and histidine (the right plate in each panel). The interaction between Smad3, which mediates transmitting signals in transforming growth factor-beta (TGF-β) signaling pathway, and

Smad ubiquitination-related factor 1 (Smurf1) was used as a positive control. Empty Gal4 bait vector pB66 / empty prey vector pP7, empty Gal4 bait vector pB66 / Gal4 Activation domain (AD)-Cib2, Gal4 DNA binding domain (DBD)-Tmc1$^{1-193}$ / empty prey vector pP7 were used as negative controls.

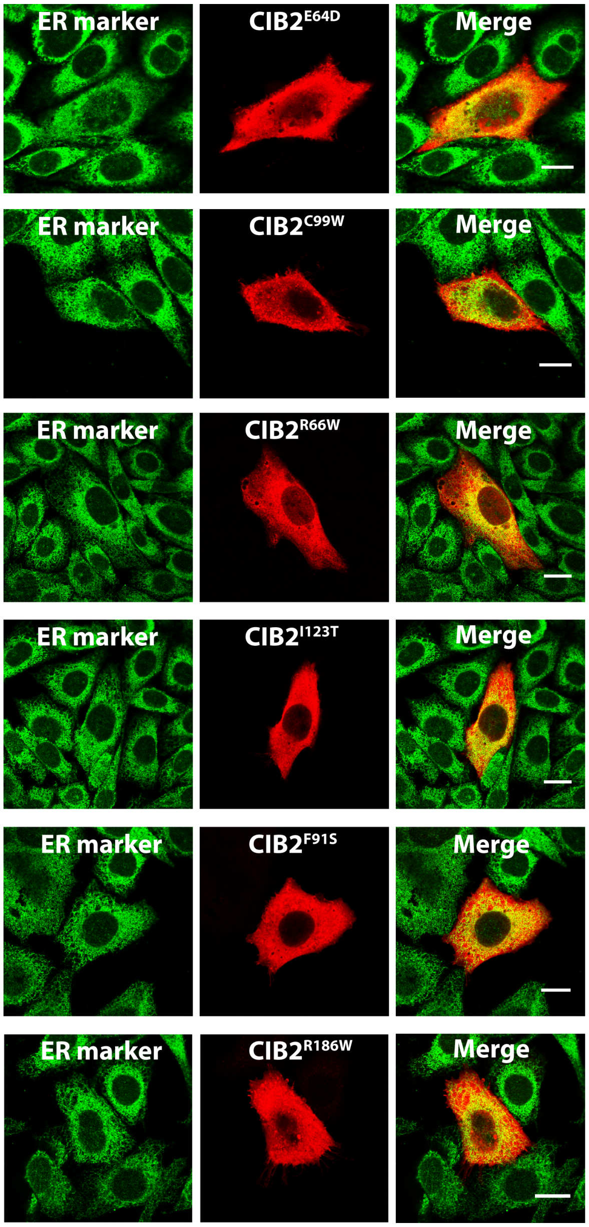

Supplementary Fig. 9 CIB2 mutations do not affect cellular distribution

Fluorescent images of CHO-K1 cells transfected with constructs expressing CIB2 mutants fused with V5 reporter and immunostained with anti-V5 and anti-KDEL, a marker for endoplasmic reticulum (ER). None of the six missense mutations affected CIB2 distribution. Scale bars: 5µm.

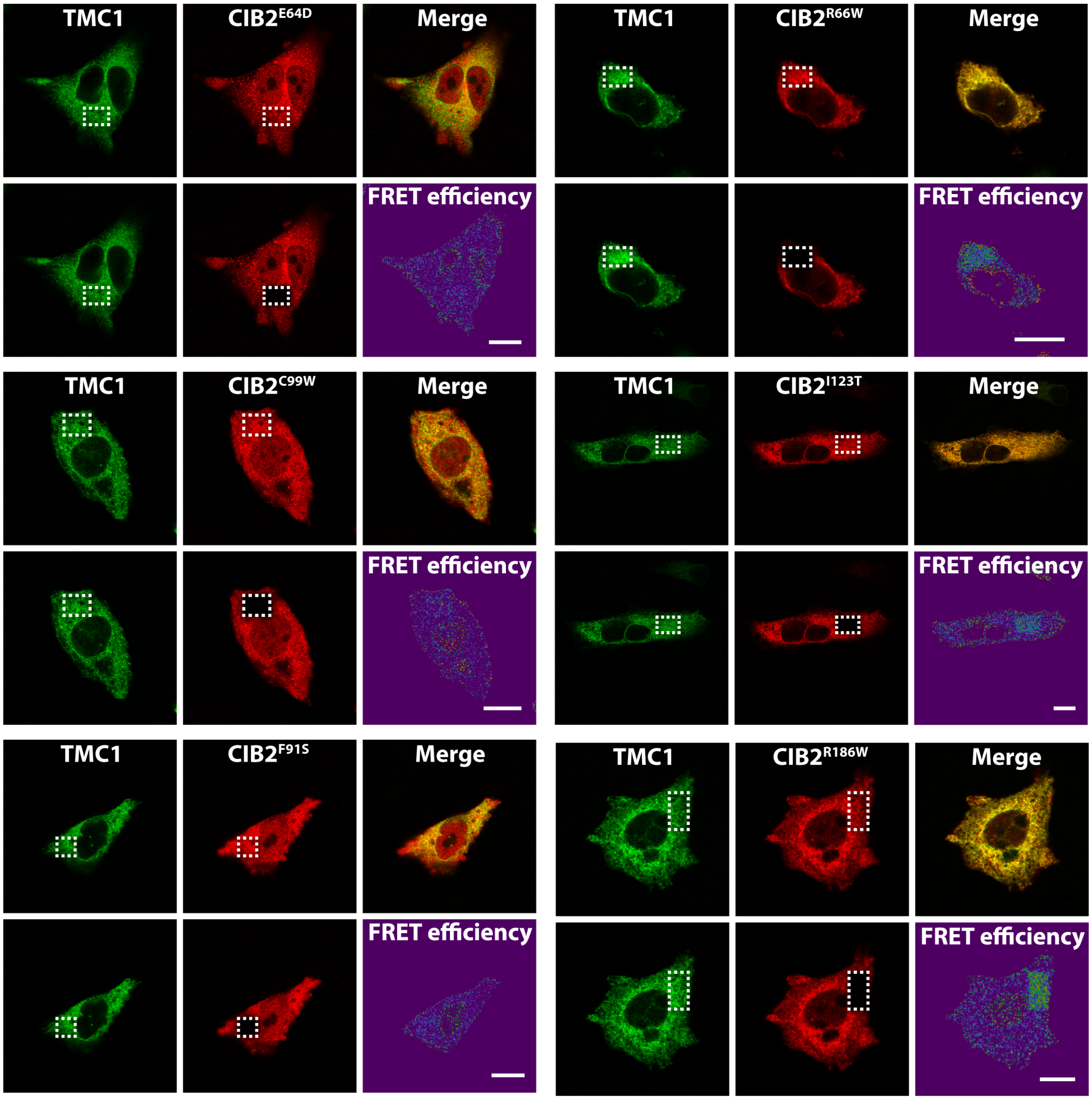

Supplementary Fig. 10 *USH1J/DFNB48* alleles of CIB2 impair its interaction with TMC1

Fluorescent images of cells co-expressing EGFP tagged human TMC1 (donor) and V5 tagged human CIB2 with deafness causing mutations (acceptor) before and after acceptor photobleaching within the indicated region (white box). Scale bars: 10 µm.

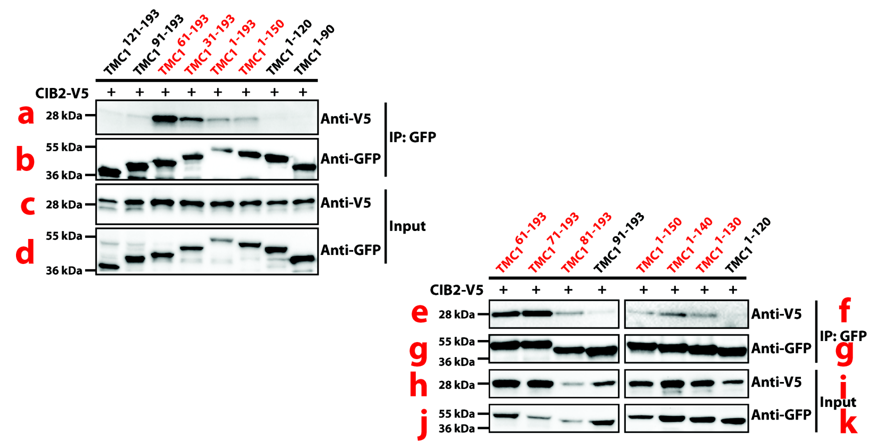

Uncropped images

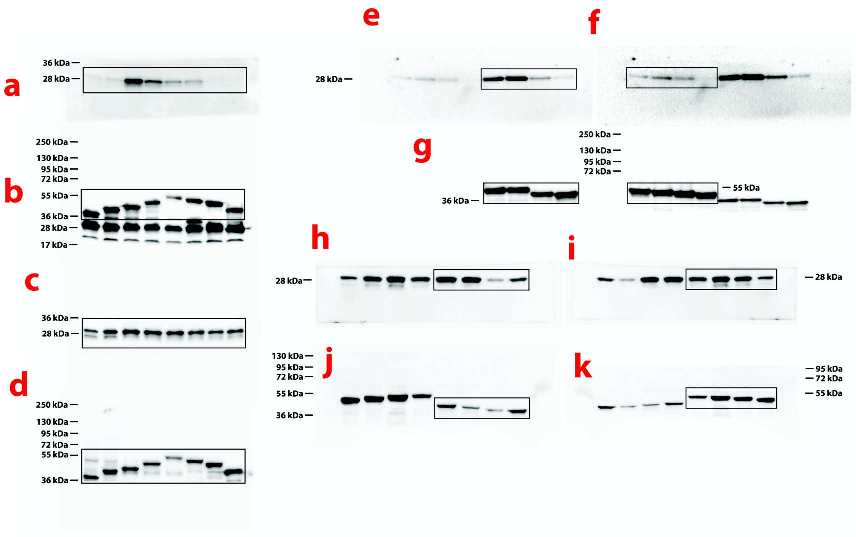

Supplementary Fig. 11 Uncropped images of films scans show in Fig. 8a

Each blot from figure 8a (top panel) was given a letter (a-k). The uncropped image of each blot is shown in the bottom panel. The black boxes indicate the cropped regions.

Gene	Acoustic Startle/PPI	Auditory Brainstem Response	Body Composition	Calorimetry	Clinical Blood Chemistry	Eye Morphology	Fertility and Viability	Glucose Tolerance Test	Grip Strength	Heart Function	Hematology	Open field	Organ Weight	Pathology and Histopathology	SHIRPA and Dysmorphology	X-ray
Cib2																

Supplementary Table 1 Phenotype heatmap of procedural data for *Cib2*$^{tm1b(EUCOMM)Wtsi}$ homozygous mice

Data are reported on the IMPC portal (http://www.mousephenotype.org/data/genes/MGI:1929293#section-associations). Background strain – C57BL/6NTac; Phenotyping centre – MRC Harwell. *P* value threshold <0.0001.

Red, Phenodeviance detected;

White, No phenodeviance detected.

Acoustic Startle/PPI -	Abnormal startle reflex; Abnormal prepulse inhibition
Auditory Brainstem Response -	Abnormal brainstem auditory evoked potential
Calorimetry -	Abnormal voluntary movement
Clinical Blood Chemistry -	Abnormal circulating HDL-cholesterol level
Heart Function -	Abnormal heart left ventricle morphology
SHIRPA and Dysmorphology -	Abnormal ear morphology; Absent startle reflex; Tremors
